# Clinical practice and outcomes in European pediatric cardiac anesthesia: A secondary analysis of the APRICOT and NECTARINE studies

**DOI:** 10.1111/aas.14585

**Published:** 2025-01-29

**Authors:** Albert Gyllencreutz Castellheim, Walid Habre, Tom Giedsing Hansen

**Affiliations:** ^1^ Department of Anesthesiology and Intensive Care Medicine, Institution of Clinical Sciences, Sahlgrenska Academy University of Gothenburg Gothenburg Sweden; ^2^ Region Västra Götaland Sahlgrenska University Hospital Gothenburg Sweden; ^3^ Faculty of Medicine University of Geneva Geneva Switzerland; ^4^ Department of Anesthesiology and Intensive Care Akershus University Hospital Lørenskog Norway; ^5^ Faculty of Medicine, Institute of Clinical Medicine Oslo University Oslo Norway

**Keywords:** cardiac surgery, congenital heart defects, data collection methods, low‐income population, outcome studies, pediatric anesthesia, pediatrics, public health

## Abstract

**Background:**

Despite advancements in surgical techniques and perioperative care, pediatric cardiac patients remain at an increased risk of adverse events. The APRICOT (2017) study aimed to establish the incidence of severe critical events in children undergoing anesthesia in Europe, while the NECTARINE (2021) study aimed to assess anesthesia practices and outcomes in neonates and infants under 60 weeks postconceptual age. Our goal was to conduct a secondary analysis of the cardiac cohorts from these two studies to determine mortality rates and other outcomes after cardiac procedures at 30 and 90 days, identify factors influencing mortality, illustrate clinical practices, and assess the methodology of the two studies.

**Methods:**

Sub‐analysis of the data from APRICOT and NECTARINE. Data representativity was assessed through a systematic categorization process. European countries were divided into four income groups based on their gross national income per capita. Subsequently, the total number of patients across all four income groups was calculated for both the Apricot and Nectarine studies, and then the specific contribution of each income group to the total population of each study was determined.

**Results:**

This analysis comprised 1016 cases (Apricot, *n* = 476 and Nectarine, *n* = 540). There was a considerable variability in clinical practice in Europe. The overall mortality rates were 0.84% (APRICOT) and 8.1% (NECTARINE). In both cohorts, substantial mortality was observed among low‐age and low‐weight infants. Stratifying the participating countries by income illustrated that the data originated from highest‐income and upper‐middle‐income European countries and were not representative of low‐income and middle‐income countries.

**Conclusions:**

In this secondary analysis of the APRICOT and NECTARINE studies, we found that fatal cases primarily occurred in low‐age and low‐weight neonates and infants.

**Editorial comment:**

This secondary analysis of the APRICOT and NECTARINE studies focused on pediatric cardiac surgical cases. Outcomes differed according to weight and age of the children, where mortality risk was higher for very young and low‐weight children.

## 1 INTRODUCTION

Congenital heart disease (CHD) is the most common birth defect worldwide, affecting approximately 1% of live births. Pediatric patients with CHD often require surgical or interventional cardiac procedures, many of which necessitate anesthesia. Additionally, acquired cardiac conditions, such as infective endocarditis or cardiomyopathy, may require interventions that involve anesthesia administration. Despite advancements in surgical techniques and perioperative care, pediatric cardiac patients remain at an increased risk of adverse events during anesthesia, including hemodynamic instability, arrhythmias, and myocardial dysfunction, and as such, they present unique challenges for anesthesia providers due to their increased susceptibility to perioperative complications. Thus, the incidence of severe critical events during anesthesia in this population remains a topic of significant clinical interest and concern.

In the last decade, two prominent prospective multicenter observational studies were performed in Europe: the incidence of severe critical events in pediatric anesthesia (APRICOT)^1^, ^2^ and the morbidity and mortality after anesthesia in early life (NECTARINE)^3^ (hereafter Apricot and Nectarine). Apricot investigated the incidence of severe critical events in children (birth to 15 years) undergoing anesthesia in Europe, aiming to elucidate differences in pediatric anesthesia practices and factors influencing anesthesia‐related morbidity and mortality, including patients with cardiac conditions. Complementing Apricot, Nectarine focused on assessing anesthesia‐associated morbidity and mortality in neonates and infants up to 60 postmenstrual weeks (approximately 5 months for full‐term infants) across Europe. Both studies employed extensive case report forms (CRFs), each containing over 110 variables across multiple sections covering patient identification, preoperative, intraoperative, and postoperative data (Data S1). The CRFs needed to be converted to electronic CRFs (eCRFs) to be reported to the study center. These multinational and multicenter European studies offered the opportunity to delve into and provide comprehensive insights into the perioperative management and outcomes of different pediatric patient categories.^4^, ^5^, ^6^, ^7^, ^8^, ^9^, ^10^ Therefore, our primary goal was to conduct a secondary analysis to determine the mortality rate following cardiac surgical procedures or catheterization within the cardiac cohorts of the Apricot and Nectarine studies. Our secondary objectives were to identify factors influencing mortality, illustrate the clinical practices, determine the 30‐day complication rates, and assess the representativeness and accuracy of the data in reflecting the actual conditions across European countries.

## 2 METHODS

### 2.1 Study design

The parent Apricot and Nectarine studies were registered in ClinicalTrials.gov under numbers NCT01878760 and NCT02350348, respectively. They were conducted between April 2014 and January 2015 and between March 2016 and January 2017 in 261 and 165 centers across 33 and 31 European countries, respectively. The current study is a sub‐analysis of the data from cardiac cohorts of the parent Apricot and Nectarine studies.

### 2.2 Participants

During each allocated enrolment period of the parent studies, children undergoing anesthesia for surgical and non‐surgical procedures were included with Apricot capturing 88% of all children under the age of 15 and Nectarine capturing almost 76% of all eligible neonates and infants up to 60 weeks postmenstrual age.

### 2.3 Variables

The methodology of the parent Apricot and Nectarine studies is described in detail in the original manuscripts (1, 2). Briefly, demographic data as well as medical history, type of the procedure, anesthesia management, and duration of the procedure were noted. To account for prematurity, the Nectarine study used postmenstrual weeks to determine the age inclusion criteria. Postmenstrual weeks were calculated by adding the chronological age (weeks since birth) and gestational age. For example, 1 year of age equals 92 postmenstrual weeks (40 weeks of gestation plus 52 weeks of a year). The Nectarine study required participants to have 60 or fewer postmenstrual weeks. By contrast, the Apricot study did not use postmenstrual weeks. Instead, it included patients based solely on chronological age, from birth up to 15 years. For Apricot, the primary outcome was severe critical events, while for Nectarine, the rate of interventions in response to a critical event during anesthesia management was the primary endpoint. The Apricot and Nectarine studies employed distinct variables to report cardiovascular instability and associated treatments. Both studies recorded 30‐day mortality rates. However, Nectarine extended its follow‐up to include 90‐day mortality and provided comprehensive outcome data at 30 days postanesthesia.

### 2.4 Data representativity

In the present study, we aimed to assess how well the data in the parent Apricot and Nectarine studies reflects actual conditions across European countries. To do this, we categorized all European countries into four income groups. This categorization was based on gross national income (GNI) per capita data from the World Bank for the year 2016, expressed in United States dollars (USD). The data were sourced from https://data.worldbank.org/. These income groups were low‐income countries (LICs) with GNI <6000 USD (*N* = 8), middle‐income countries (MICs) with GNI 7000–13,000 USD (*N* = 8), upper‐middle‐income countries (UMICs) with GNI 14,000–32,000 USD (*N* = 12), and highest‐income countries (HICs) with GNI >33,000 USD (*N* = 16). In the APRICOT parent study, 27 countries participated, including 13 HICs, 6 UMICs, 5 MICs, and 3 LICs countries. In the Nectarine parent study, 29 countries participated, encompassing 13 HICs, 7 UMICs, 6 MICs, and 3 LICs countries. For each parent study, we calculated the total number of patients across all four income groups and the specific contribution of LICs and MICs groups of countries to the total populations of each parent study. The mapping of European countries based on their 2022 GNI per capita data reveals consistent income categories across the continent. The average increase in GNI per capita from 2016 to 2022 across all income categories (LICs + MICs + UMICs + HICs) is approximately 33%. Despite the overall increase in GNI per capita from 2016 to 2022, the relative income differences between the four groups have remained largely constant.

### 2.5 Statistical analysis

Data concerning patients who underwent cardiac surgeries or catheterization were retrieved from the Apricot and Nectarine datasets, which were extracted from Open Clinica and formatted in Excel. The R programming language (https://www.r-project.org/) (R Core Team (2019). R: A language and environment for statistical computing. R Foundation for Statistical Computing, Vienna, Austria), was used for the extraction and organization of this data. The information is presented using descriptive statistics: frequencies and percentages for categorical variables, means with standard deviations for normally distributed continuous variables, and medians with interquartile ranges for non‐normally distributed data. Additionally, logistic regression analyses were performed using R, with the inclusion of specific predictor variables deemed relevant for each model.

## 3 RESULTS

### 3.1 Patient characteristics

In the present study, the analysis was performed on 1016 cases (Apricot, *n* = 476, and Nectarine, *n* = 540). This represents 1.5% and 8.2% of the total procedures recorded in these studies. Table 1 depicts patient demographics, showing Apricot including a broad age and weight spectrum, with some over 40 kg, while Nectarine is limited to under 10 kg.

**TABLE 1:** Demography.

Variables	Apricot		Nectarine	
	Surgery (*n* = 245)	Catheterization (*n* = 231)	Surgery (*n* = 439)	Catheterization (*n* = 101)
Female, *n* (%)^a^	105 (42.9)	114 (49.4)	192 (43.7)	44 (43.6)
Male	140, (57.1)	117, (50.6)	247 (56.3)	57 (56.4)
Age, median [IQR]	52 [190]	208 [468]	41 [9.5]	41 [10]
≤60 (PM Weeks)	115 (47.7)	41 (18.0)	439 (100)	101 (100)
>60 to ≤92 (PM Weeks)	6 (2.5)	4 (1.8)	–	–
>1–3 years	50 (20.7)	58 (25.4)	–	–
>3–10 years	39 (16.2)	73 (32.0)	–	–
>10–15 years	31 (12.9)	52 (22.8)	–	–
Missing, *n*	4	3		
Weight, kg, (mean ± SD)	15.5 ± 17.4	23.9 ± 19.8	3.4 ± 1.3	3.9 ± 1.2
≤3	24 (9.8)	5 (2.2)	149 (34.1)	25 (24.8)
>3–10	116 (47.5)	57 (24.8)	288 (65.9)	76 (75.2)
>0–20	50 (20.5)	70 (30.4)	–	–
>20–40	28 (11.5)	55 (23.9)	–	–
>40	26 (10.7)	43 (18.7)	–	–
Missing, *n*	1	1	2	0
Preoperative respiratory treatment				
Patient intubated	–	–	137 (31.2)	17 (16.8)
Missing, *n*	–	–	0	0
Length of surgery/procedure, min, (mean ± SD)	176.7 ± 111.5	85.6 ± 54.9	230.9 ± 145.6	134.8 ± 156.6
≤90	56 (23.2)	148 (64.4)	83 (19.3)	38 (38.4)
>90–180	82 (34.0)	74 (32.2)	100 (23.3)	44 (44.4)
>180–360	89 (37.0)	7 (3.0)	181 (42.1)	16 (16.2)
>360	14 (5.8)	1 (0.4)	66 (15.3)	1 (1.0)
Missing, *n*	4	1	9	2
Postanesthesia hospital stay, days, (mean ± SD)	10.1 ± 11.2	2.1 ± 3.9	15.7 ± 14.4	13.5 ± 16.6
≤7	109 (53.2)	195 (95.1)	69 (26.6)	40 (56.3)
>7–14	54 (26.3)	7 (3.4)	90 (34.7)	5 (7.0)
>14–21	24 (11.7)	1 (0.5)	52 (20.1)	8 (11.3)
>21–30	14 (6.8)	1 (0.5)	25 (9.7)	10 (14.1)
>30	4 (2.0)	1 (0.5)	23 (8.9)	8 (11.3)
Missing, *n*	23	8	120	18

Abbreviation: PM Weeks, postmenstrual weeks.

^a^ Data are presented as count (percentage) unless otherwise specified.

### 3.2 Apricot outcomes of surgery and catheterization at 30 days

Table 2 outlines the 30‐day postanesthesia outcomes from Apricot for different age and weight groups. Infants <60 PM Weeks had a 1.9% surgery mortality rate while the mortality rate for catheterization was 2.6%. Most were discharged (67.9% surgery, 78.9% catheterization), though some remained hospitalized (17.0% surgery, 13.2% catheterization). No deaths were reported in >60 to ≤92 PM Weeks and 1–3 years age groups, with high discharge rates (83.3% for surgery, 100% for catheterization in 1–3 years).

**TABLE 2:** Apricot outcomes of surgery and catheterization at 30 days.

A: Outcomes stratified by age	Age at surgery					Age at catheterization				
Outcome variables	<60 (PM Weeks)	>60 to ≤92 (PM Weeks)	>1–3 years	>3–10 years	>10–15 years	<60 (PM Weeks)	>60 to ≤92 (PM Weeks)	>1–3 years	>3–10 years	>10–15 years
Dead	2 (1.9)	0 (0)	0 (0)	0 (0)	1 (3.3)	1 (2.6)	0 (0)	0 (0)	1 (1.4)	0 (0)
Discharged to										
Home	72 (67.9)	2 (50)	40 (83.3)	33 (84.6)	27 (90.0)	30 (78.9)	3 (75.0)	52 (100)	68 (97.1)	49 (98.0)
Acute center	9 (8.5)	2 (50)	0 (0)	2 (5.1)	0 (0)	2 (5.3)	0 (0)	0 (0)	0 (0)	0 (0)
Convalescent	5 (4.7)	0 (0)	4 (8.3)	2 (5.1)	1 (3.3)	0 (0)	1 (25.0)	0 (0)	0 (0)	0 (0)
Still in hospital	18 (17.0)	0 (0)	4 (8.3)	2 (5.1)	1 (3.3)	5 (13.2)	0 (0)	0 (0)	1 (1.4)	1 (2.0)

B: Outcomes stratified by weight	Weight at surgery					Weight at catheterization				
Outcome variables	<3 kg	>3–10 kg	>10–20 kg	>20–40 kg	>40 kg	<3 kg	>3 to 10 kg	>10 to 20 kg	>20 to 40 kg	>40 kg
Dead	1 (5.0)	1 (0.9)	0 (0)	0 (0)	1 (3.8)	1 (20.0)	1 (2.1)	0 (0)	0 (0)	0 (0)
Discharged to										
Home	10 (50.0)	76 (69.7)	42 (87.5)	24 (88.9)	23 (88.5)	2 (40.0)	41 (85.4)	66 (98.5)	51 (98.1)	42 (100)
Acute center	2 (10.0)	10 (9.2)	2 (4.2)	0 (0)	0 (0)	1 (20.0)	1 (2.1)	0 (0)	0 (0)	0 (0)
Convalescent	1 (5.0)	7 (6.4)	2 (4.2)	1 (3.7)	1 (3.8)	0 (0)	1 (2.1)	0 (0)	0 (0)	0 (0)
Still in Hospital	6 (30.0)	15 (13.8)	2 (4.2)	2 (7.4)	1 (3.8)	1 (20.0)	4 (8.3)	1 (1.5)	1 (1.9)	0 (0)

*Note*: Data are presented as count (percentage) unless otherwise specified.

Abbreviation: PM Weeks, postmenstrual weeks.

### 3.3 Nectarine outcomes of surgery and catheterization at 30 and 90 days

Table 3 shows Nectarine's follow‐up and mortality postanesthesia at 30 and 90 days, based on patient weight. At 30 days, follow‐ups were 95% for surgery and 97% for catheterization but dropped by 90 days to around 70%. Patients under 3 kg had higher mortality at 30 days (13.6% for surgery, 16.7% for catheterization) than those over 3 kg (5.9% for surgery, 6.8% for catheterization). Deaths between 30 and 90 days were infrequent (1.0% for surgery under 3 kg, 1.5% for over 3 kg). By 30 days, 20% of lower‐weight surgical patients were still in ICU, higher than the 9.2% of higher‐weight patients; for catheterization, the rates were 4.2% for under 3 kg and 5.4% for over 3 kg. Lower‐weight patients had longer ICU stays and more hospital transfers. Brain/CNS complications occurred in 17.3% of lower‐weight surgery patients, while higher‐weight patients had more surgical and cardiovascular complications. By day 90, re‐admissions were 26.8% higher for catheterization patients >3 kg.

**TABLE 3:** Nectarine outcomes at 30 and 90 days (age <60 PM Weeks).

	Surgery	Catheterization	Surgery		Catheterization	
	All patients	All patients	<3 kg	>3–10 kg	<3 kg	>3–10 kg
Outcomes variables at 30 days						
Follow‐up done	416 (95.0)	98 (97.0)	141 (94.6)	273 (95.1)	24 (96.0)	74 (97.4)
Death	35 (8.4)	9 (9.2)	19 (13.6)	16 (5.9)	4 (16.7)	5 (6.8)
Still in ICU	53 (12.8)	5 (5.1)	28 (20.0)	25 (9.2)	1 (4.2)	4 (5.4)
Discharged to another hospital	48 (11.6)	7 (7.1)	30 (21.4)	18 (6.6)	2 (8.3)	5 (6.8)
Total days in PICU/NICU (mean ± SD)	13.8 ± 12.1	12.4 ± 10.9	18.9 ± 14.2	11.1 ± 9.9	14.7 ± 11.5	11.1 ± 10.6
Brain/CNS complication	30 (13.5)	0 (0)	14 (17.3)	16 (11.3)	0 (0)	0 (0)
Surgical complication	75 (33.8)	12 (40)	25 (30.9)	50 (35.5)	2 (22.2)	10 (47.6)
Cardiovascular complication	163 (73.4)	19 (63.3)	52 (64.2)	111 (78.7)	6 (66.7)	13 (61.9)
Renal insufficiency	44 (19.8)	5 (16.7)	17 (21.0)	27 (19.1)	2 (22.2)	3 (14.3)
Outcomes variables at 90 days						
Follow‐up done	307 (69.9)	72 (71.3)	99 (66.4)	206 (71.5)	16 (64.0)	56 (73.7)
Death⸺Day 30–90	4 (1.3)	0 (0)	1 (1.0)	3 (1.5)	0 (0)	0 (0)
Hospital re‐admission until Day 90	58 (19.1)	17 (23.6)	16 (16.3)	42 (20.6)	2 (12.5)	15 (26.8)

*Note*: Data are presented as count (percentage).

### 3.4 Clinical practice data

The outlines of the utilization patterns of *blood and fluid therapy, hemostatic agents, as well as vasoactive and inotropic agents* are shown in the Data S1.

### 3.5 Regression analyses of mortality

In a logistic regression analysis of the Nectarine study's surgical cohort, consisting of 439 patients with 35 fatalities, the duration of surgery was identified as the only significant predictor of mortality, with longer operations linked to a higher likelihood of death. Other factors, including age, RBC count, weight, gender, and intubation status, were not significantly associated with mortality. A subsequent combined analysis of the surgery and catheterization groups, totaling 540 patients and 44 deaths, confirmed that surgery length was a consistent predictor of mortality, with the impact of patient weight on mortality bordering on statistical significance (*p* = .050).

In the Apricot study, conventional logistic regression analysis was precluded due to the limited rate of the dependent variable (mortality). Therefore, an alternative approach utilizing Firth's Bias‐Reduced Penalized‐Likelihood Logistic Regression was employed. The presence of an excessively high correlation between two independent variables, namely weight and age, posed a risk of multicollinearity; consequently, it necessitated the separate inclusion of these predictors in the regression models. Analytical outcomes indicated that none of the predictor variables under consideration exerted a statistically significant influence on mortality rates (Data S1).

### 3.6 Data representability

The LICs contributed 11.11% and the MICs 11.03% to the total study population in the primary main Apricot study. The LICs group contributed 4.02% and the MICs group 10.55% to the total study population in the primary main Nectarine study. Hence, the total contribution of LICs and MICs combined was approximately 22 and 14, respectively (data not shown).

## 4 DISCUSSION

This secondary analysis of the Apricot and Nectarine cohorts provides insight into pediatric cardiac anesthesia practices and patient outcomes following cardiac surgery and/or catheterization in Europe. Pediatric patients with cardiac conditions present unique challenges during anesthesia, including hemodynamic instability and altered response to anesthetic agents. Factors such as the complexity and duration of cardiac surgical procedures, comorbidities, and procedural characteristics play a pivotal role in patient outcomes. In our study, we observed an overall mortality rate of 0.84% in the Apricot study and 8.1% in the Nectarine study. Although we could not calculate the standardized mortality rate (SMR) due to the characteristics of our study, the observed mortality rates align with existing US‐based literature on postoperative mortality in pediatric patients undergoing congenital heart surgery.^11^, ^12^

A key finding of our study is that mortality rates following cardiac management in children with **CHD** in Apricot and Nectarine are predominantly concentrated in a specific age group: neonates and infants. Data extracted from Apricot show that outcomes appear to improve with increasing age and weight (Table 2). Specifically, higher rates of discharge to home and lower mortality rates are observed. Nectarine data (Table 3) highlight differences in complication rates between surgery and catheterization procedures within the first 30 days postprocedure, particularly in children >3 kg. The immediate postprocedure complications are more common in the surgery group; however, catheterization patients (especially those over 3 kg) exhibit higher re‐admission rates at 90 days.

The Apricot and Nectarine studies primarily focused on investigating severe critical events in pediatric anesthesia and assessing postanesthesia outcomes. Consequently, their CRFs did not capture information on diagnoses, procedural indications, or technical aspects of interventions. The CRFs were neither procedure‐specific nor exhaustively detailed for individual procedures. For example, in cardiac cases, data on cardiopulmonary bypass utilization and related metrics were not collected. Notably, in CHDs, where complexity and disease burden are largely determined by specific diagnoses, this diagnostic information was not recorded in either study's CRFs. Therefore, we used the length of surgery as a surrogate marker to estimate the complexity of CHDs. Using logistic regression analysis on the surgical cohort from Nectarine, we identified that the duration of surgery was the sole significant predictor of mortality. Prolonged surgical durations may indicate more intricate procedures required to address complex cardiac anomalies. However, using the length of surgery as a surrogate for complexity has limitations. This approach assumes consistency across hospitals and surgical teams, overlooking important variables such as surgical skill, available resources, institutional protocols, and patient‐specific factors. Longer surgeries may not always indicate greater complexity; they could result from complications, unforeseen challenges, or meticulous surgical approaches that might actually improve outcomes. Therefore, while surgical duration provides valuable insights, it should be interpreted cautiously within the broader context of each case and healthcare setting.

Another significant finding from this analysis is the identification of a relatively high rate of postoperative cardiovascular complications in neonates and small infants, both after cardiac surgery and catheterization. While this sub‐analysis does not establish a direct correlation between this high rate and the occurrence of, for example, renal insufficiency or cerebral complications, our results highlight the complexity of neonatal cardiac surgery and its associated high morbidity and mortality.

Preoperative invasive mechanical ventilation, especially on the day of surgery, has been shown to correlate with prolonged postoperative hospital stays in both children and neonates.^13^

Focusing on neonates, this retrospective study found that preoperative use of high‐flow nasal oxygen via a cannula or noninvasive ventilation increased operative mortality, with no significant association with postoperative complications.^13^ Interestingly, in our regression model of the Nectarine's surgical cohort, preoperative ventilation had a negative effect (Coef. = −1.2) on the outcome and was close to being significant (*p* = .0966), suggesting preoperative intubation and ventilation were associated with lower odds of the outcome, with an odds ratio of 0.3.

Key factors distinguishing clinical practices among centers include fluid balance management, blood transfusion protocols, and the type and extent of vasopressor and inotrope usage.

Concerning fluid balance, neither study included data on fluid balance. In the context of pediatric cardiac surgery, fluid overload poses a significant clinical challenge and is associated with poor outcomes.^14^, ^15^, ^16^ As shown in the Data S1, patients frequently required substantial volumes of infusions, including medications and blood products. The received fluid is concurrent with the fluid retention provoked by surgical stress, cardiopulmonary bypass, and the impairment of cardiac and, consequently, renal function. Similarly to what has been previously reported,^17^ there is a huge variance in postsurgery blood and fluid management techniques. In a study from 2020 based on the STS Congenital Heart Surgery Database, 76.5% of patients on cardiopulmonary bypass received non‐autologous blood transfusions, with the highest rates in neonates (91.2%) and infants (89.1%), and the lowest in adults (46.5%).^18^ These results align with the findings from the Apricot study concerning the red blood cell (RBC) transfusion. However, within the surgical cohort of the Nectarine study, only 46.7% of patients were administered RBC transfusions. Interestingly, the percentage of children receiving platelets was higher in Apricot than in Nectarine. This finding may be related to the period when the studies were conducted and before promoting fibrinogen in neonates for preventing postoperative bleeding.^19^

In pediatric cardiac surgery, the administration of vasopressors and inotropes plays a critical role in preventing low cardiac output syndrome. The vasoactive‐inotropic score serves as a valuable metric for assessing the requisite level of cardiac support, with higher scores generally associated with less favorable postoperative outcomes in infants.^20^ The Apricot dataset indicates that vasopressors were administered in up to 22% of cases. However, in neonates and infants, the most frequently utilized agents for managing intraoperative hemodynamic instability were epinephrine, milrinone, phenylephrine, and dopamine. While inter‐study comparisons present challenges due to variations in patient demographics, surgical procedures, and institutional protocols, our findings underscore the substantial variability in clinical practices throughout Europe.

*One major focus of our study is to critically examine the methodology employed in the Apricot and Nectarine studies. While these studies are 7–10 years old, the methodological challenges they reveal remain relevant today such as selection bias from voluntary participation, inconsistent consent requirements, reliance on self‐reporting, and underrepresentation of low‐ and middle‐income countries*. To assess the representativeness of our data for all European countries, we needed to investigate the representativeness of the main Apricot and Nectarine data for all European countries.

Our analysis of data from the Apricot and Nectarine studies indicated that participation and data load from LICs and MICs were underrepresented. Specifically, the data load from these countries was only 22% for the Apricot study and 14% for the Nectarine study. European countries are generally skewed towards higher income categories, with fewer countries in the LICs and MICs groups. This naturally results in a skewed data load, with more data coming from UMICs and HICs.

Economic and research infrastructures in low‐income LICs and MICs often impede full participation in large‐scale clinical studies, making proportional representation challenging. Consequently, studies like Apricot and Nectarine may not be optimal for acquiring accurate and sufficient data from these regions. Physician participation, crucial for such research projects, is primarily influenced by resource availability, which is frequently limited in LICs and MICs. Additional factors constraining research in these countries include regulatory environments, leadership support, time and workload pressures, financial incentives, and professional development opportunities.

Research culture, infrastructure, and resources significantly influence participation in large‐scale studies. Factors such as research experience, study design, patient demographics, consent processes, and interdepartmental collaboration also play crucial roles. These elements particularly impact the ability of physicians and institutions in LICs and MICs to engage in extensive research projects like Apricot and Nectarine. The time‐intensive nature of completing CRFs and converting them to eCRFs, in conjunction with reporting, coupled with existing workload challenges in LICs and MICs, presents a substantial barrier to participation. To address these issues and ensure accurate, comprehensive data collection from these regions, it may be necessary to conduct targeted research focusing specifically on LICs and MICs, thereby addressing their unique challenges and ensuring data relevance and reliability. In addition to this targeted research approach aimed at identifying the rate of complications and their etiologies, as well as the rates of morbidity and mortality, there is also a need for allocated resources and targeted training in these countries. There is satisfactory evidence that training,^21^ collaboration,^22^ and standardization initiatives, such as the Congenital Heart Surgeons' Society's protocol standardization effort,^23^ have led to significantly improved outcomes.

## 5 LIMITATIONS

Our study has several limitations. First, the Apricot and Nectarine studies were not specifically designed to evaluate postoperative outcomes following congenital heart surgery or catheterization procedures, which necessitates careful interpretation of the generalizability of these results.

Second, integrating results from these two distinct study populations presents inherent methodological challenges, which may further complicate the extrapolation of the findings. Third, as cross‐sectional observational studies, Apricot and Nectarine have typical constraints such as the inability to deduce causality or chronological associations. Fourth, participation was voluntary and inconsistent regarding parental consent requirements, increasing the risk of creating selection bias. Fifth, the reliance on self‐reporting may have influenced selection bias and limited the breadth of data collected.

Sixth, the focus on responses to critical events, excluding preventive measures, minimizes the scope for evaluating the quality of anesthesia care in pediatrics and infants. Furthermore, while surgery duration provides valuable insights, it should be considered with caution as a surrogate for complexity, as it may overlook factors such as surgical team experience, available resources, and patient‐specific variables that can influence operative time independently of the procedure's inherent complexity.

In addition, the data primarily originating from European UMICs and HICs may not accurately reflect the conditions in European LICs and MICs. This heterogeneity could significantly affect the outcome measures, limiting the generalizability of our findings to the entire European population. The study's design may also introduce selection bias, as the data are not representative of all European countries. This bias could skew the mortality figures and other outcome measures, making them less reliable for broader applications. The mortality figures are influenced by both economic and non‐economic factors. While economic differences between UMICs/HICs and LICs/MICs play a crucial role, other factors such as healthcare infrastructure, access to care, and population health also significantly impact the outcomes. This complexity underscores the need for cautious interpretation of our results. Finally, our study does not represent the entire European population, and this limitation highlights the need for future studies to include data from a more diverse range of countries, including LICs and MICs. Such studies would help to provide a more comprehensive understanding of the real situation and enhance the generalizability of the findings.

In summary, we observed an overall mortality rate of 0.84% in the Apricot study and 8.1% in the Nectarine study, with mortality primarily occurring in low‐age and low‐weight neonates and infants. We also found that perioperative critical events and comorbidities are common.

These findings are primarily based on data from UMICs and HICs and therefore do not reflect the conditions in LICs and MICs, nor do they represent Europe as a whole.

Given the limitations, this paper provides valuable insights for improving future research design in this field and aims to serve as a critical baseline for developing more robust and inclusive research methodologies in pediatric cardiac anesthesia, addressing the fundamental challenges in conducting representative, multicenter studies across diverse European healthcare systems. Future research should focus on targeted programs in low‐income European nations to address gaps in pediatric cardiac care. Additionally, randomized controlled trials (RCTs) are needed to include diverse patient populations and investigate new therapeutic strategies, such as advanced surgical techniques and novel pharmacotherapies. By addressing these gaps, future studies can provide a more comprehensive understanding of pediatric cardiac care and improve outcomes across all European countries.

### 5.1 Conclusions

In this secondary analysis of the APRICOT and NECTARINE studies, we found that fatal cases primarily occurred in low‐age and low‐weight neonates and infants.

## AUTHOR CONTRIBUTIONS

Walid Habre provided the data for the study. All authors (Albert Gyllencreutz Castellheim, Walid Habre, Tom Giedsing Hansen) equally contributed to the study design, data analysis, interpretation of the results, drafting of the manuscript, and critical revisions. All authors have reviewed and approved the final version of the manuscript and agree to be accountable for all aspects of the work.

## FUNDING INFORMATION

No funding was received for this research.

## CONFLICT OF INTEREST STATEMENT

The authors declare no conflicts of interest.

## Supporting information


**Data S1.** Apricot and Nectarine clinical practices, regression models for mortality, and case report forms.

## Data Availability

The data that support the findings of this study are available on request from the corresponding author. The data are not publicly available due to privacy or ethical restrictions.
