# Supplementary material for: Clinical practice and outcomes in European pediatric cardiac anesthesia: A secondary analysis of the APRICOT and NECTARINE studies
Source: Acta Anaesthesiol Scand. 2025 Jan 29;69(3):e14585. doi: 10.1111/aas.14585 (PMC11780212; doi:10.1111/aas.14585)
Supplement: Supplementary file 1 — Data S1. Apricot and Nectarine clinical practices, regression models for mortality, and case report forms. [file AAS-69-0-s001.pdf]

Apricot–administered Fluids, Blood, and blood products

Age	Crystalloids	Albumin	Other Colloids	FFP	RBC	Platelets	Fibrinogen
**At Surgery**							
≤60(PM Weeks)	105 (84.7)	26 (22.6)	24 (20.9)	40 (49.4)	77 (96.3)	23 (28.8)	11 (13.8)
>60 to ≤ 92 (PM Weeks)	4 (80.0)	3 (60.0)	0 (0)	1 (20.0)	4 (80.0)	2 (40.0)	0 (0)
≥1 to 3 years	49 (92.5)	6 (12.0)	7 (14.0)	11 (42.3)	24 (92.3)	10 (38.5)	4 (15.4)
>3 to10 years	36 (90.0)	4 (10.5)	5 (13.2)	11 (68.8)	14 (87.5)	7 (43.8)	2 (12.5)
>10 to 15 years	31 (100)	2 (6.3)	4 (12.5)	3 (37.5)	7 (87.5)	2 (25.0)	3 (37.5)
**At Catheterization**							
≤ 60 (PM Weeks)	34 (82.9)	2 (4.9)	2 (4.9)	–	–	–	–
>60 to ≤ 92 (PM Weeks)	4 (100)	0 (0)	0 (0)	1 (100)	0 (0)	0 (0)	0 (0)
≥1 to 3 years	51 (92.7)	1 (1.9)	0 (0)	0 (0)	1 (100)	0 (0)	0 (0)
>3 to10 years	71 (97.3)	1 (1.4)	1 (1.4)	0 (0)	2 (66.7)	2 (66.7)	1 (33.3)
>10 to 15 years	52 (100)	0 (0)	0 (0)	–	–	–	–

Data are presented as count (percentage).

Nectarine: Fluids, blood, blood products, and other agents administered to treat cardiovascular instability

	**Crystalloids** (>20 ml kg^{-1})	**Albumin** (>10 ml kg^{-1})	**Other colloids** (>10 ml kg^{-1})	**FFP** Yes	**RBC** Yes	**Platelets**	**Fibrinogen**	**Tranexamic acid**	**Prothrombin complex**	**Factor VII**
**Surgery**	65 (36.1)	38 (21.1)	21 (11.7)	62 (34.4)	84 (46.7)	6 (22.2)	0 (0)	0 (0)	0 (0)	0 (0)
**Catheterization**	17 (63.0)	4 (14.8)	2 (7.4)	0 (0)	3 (11.1)	0 (0)	0 (0)	0 (0)	0 (0)	0 (0)

Data are presented as count (percentage).

The use of vasoactive and inotropic drugs for hemodynamic instability in Apricot and Nectarine

	Apricot	Nectarine									
	Vasopressor	Ephedrine	Phenylephrine	Norepinephrine	Dopamine	Nitroglycerine	Dobutamine	Epinephrine	Milrinone	Levosimendan	Nitroprusside
**Surgery**											
≤90 min	9 (47.4)	1 (3.8)	2 (7.7)	6 (23.1)	5 (19.2)	0 (0)	2 (7.7)	10 (38.5)	4 (15.4)	1 (3.8)	0 (0)
>90 to 180 min	9 (31.0)	3 (8.3)	4 (11.1)	8 (22.2)	8 (22.2)	1 (2.8)	2 (5.6)	8 (22.2)	6 (16.7)	0 (0)	0 (0)
>180 to 360 min	22 (31.9)	13 (14.0)	30 (32.3)	20 (21.5)	20 (21.5)	1 (1.1)	3 (3.2)	30 (32.3)	26 (27.7)	2 (2.2)	1 (1.1)
>360 min	5 (23.8)	2 (5.3)	5 (13.2)	12 (31.6)	9 (23.7)	1 (2.6)	2 (5.3)	24 (63.2)	18 (47.4)	2 (5.3)	1 (2.6)
**Catheterization**											
≤90 min	4 (44.4)	5 (35.7)	2 (14.3)	2 (14.3)	0 (0)	0 (0)	0 (0)	3 (21.4)	0 (0)	0 (0)	0 (0)
>90 to 180 min	8 (47.1)	1 (6.7)	5 (33.3)	2 (13.3)	1 (6.7)	0 (0)	1 (6.7)	2 (13.3)	2 (13.3)	0 (0)	0 (0)
>180 to 360 min	1 (33.3)	0 (0)	3 (50)	1 (16.7)	1 (16.7)	0 (0)	0 (0)	1 (16.7)	1 (16.7)	0 (0)	0 (0)
>360 min	–	–	–	–	–	–	–	–	–	–	–

Data are presented as count (percentage).

Regression models for Nectarine and Apricot

Nectarine

1 Nectarine - Regression results of surgery patients

Variable	Coefficient (log odds)	Estimated Standard Error	z-statistic	*P* value	OR	95%CI Lower	95%CI Upper
Intercept	-1.0	3.9	-0.2	0.8049	0.4	0.0001	473.6
Age (weeks)	-0.03	0.08	-0.4	0.6967	1.0	0.8	1.2
RBC	0.01	0.5	0.02	0.9847	1.0	0.4	2.8
Weight	-0.56	0.3	-1.7	0.0886	0.6	0.3	1.0
Sex	0.9	0.5	1.7	0.0959	2.4	0.9	7.1
Surgery Length (min)	0.005	0.002	3.4	0.0007	1. 005	1.002	1.008
Patient intubated	-1.2	0.7	-1.7	0.0966	0.3	0.07	1.1

The model involved 439 patients with 35 deaths

2 Nectarine - Regression results of surgery and catheterization patients

Variable	Coefficient (log odds)	Estimated Standard Error	z-statistic	*P* value	OR	95%CI Lower	95%CI Upper
Intercept	-2.7	3.8	-0.7	0.4795	0.07	0.00002	70.5
Age (weeks)	0.01	0.08	0.2	0.8752	1.0	0.9	1.2
RBC	-0.09	0.5	-0.2	0.8510	0.9	0.4	2.3
Weight	-0.6	0.3	-2.0	0.0509	0.5	0.3	0.9
Sex	0.7	0.5	1.4	0.1670	1.9	0.8	5.0
Surgery Length (min)	0.005	0.001	3.2	0.0012	1.005	1.002	1.007
Patient intubated	-0.6	0.6	-1.1	0.2749	0.5	0.2	1.5

The model involved 540 patients and 44 deaths

Apricot

1 Apricot - Regression results of surgical patients, not including Weight

Variable	Coefficient (log odds)	Estimated Standard Error	*P* value	χ2	Odds ratio	95%CI Lower	95%CI Upper
Intercept	-3.1	2.5	0.2877	1.1	0.04	0.00004	22.1
Age (weeks)	0.0007	0.003	0.8404	0.04	1.0007	0.9	1.006
RBC	-1.3	1.5	0.4981	0.5	0.3	0.01	42.7
Sex	-0.5	1.4	0.7730	0.08	0.6	0.004	17.7
Surgery Length (min)	0.006	0.004	0.2269	1.5	1.006	1.0	1.01

2 Apricot - Regression results of surgical patients, not including Age

Variable	Coefficient (log odds)	Estimated Standard Error	*P* value	χ2	Odds ratio	95%CI Lower	95%CI Upper
Intercept	-2.9	2.6	0.3264	1.0	0.05	0.00005	29.1
Weight	0.01	0.03	0.8048	0.06	1.01	0.1	1.08
RBC	-1.2	1.5	0.5071	0.4	0.3	0.01	43.8
Sex	-0.6	1.5	0.7033	0.1	0.5	0.004	13.9
Surgery Length (min)	0.005	0.003	0.2595	1.3	1.005	1.0	1.01

3 Apricot - Regression results of all surgery and catheterization patients, not including Weight

Variable	Coefficient (log odds)	Estimated Standard Error	*P* value	χ2	Odds ratio	95%CI Lower	95%CI Upper
Intercept	-4.4	2.4	0.0717	3.2	0.01	0.00001	1.4
Age (weeks)	0.0007	0.002	0.7775	0.08	1.0007	1.0	1.005
RBC	-0.8	1.4	0.6391	0.2	0.5	0.04	61.9
Sex	0.5	1.0	0.7049	0.1	1.6	0.1	25.3
Surgery Length (min)	0.005	0.004	0.2725	1.2	1.005	1.0	1.01

4 Apricot - Regression results of all surgery and catheterization patients, not including Age

Variable	Coefficient (log odds)	Estimated Standard Error	*P* value	χ2	Odds ratio	95%CI Lower	95%CI Upper
Intercept	-4.1	2.4	0.1005	2.7	0.02	0.00001	2.2
Weight	0.004	0.03	0.9257	0.009	1.004	0.7	1.1
RBC	-0.8	1.4	0.6548	0.2	0.5	0.04	64.3
Sex	0.4	1.0	0.7627	0.1	1.4	0.1	23.7
Surgery Length (min)	0.004	0.003	0.3143	1.0	1.004	1.0	1.01

This Coversheet intends to help site staff and investigator linking local patient data to the study specific study patient code and this sheet is used to facilitate the task to the investigators, and can be filled to your convenience. After completing follow-up, this Coversheet should be saved apart from the CRF and filed separately in a secure place. The information on this coversheet will NOT be collected in the CRF. It is for local use ONLY.

APRICOT

Confidential Patient Identification Coversheet

- Patient Hospital/local Identification Number (handwritten or sticker):

- Patient name: Last ____________________ First ________________________

- OpenClinica Study Subject ID: |__|__|__| - |__|__|__| - |__|__|__|

- Date of Anaesthesia: _ _ /_ _ _ /_ _ _ _ (dd/Mmm/yyyy)
- Type of procedure: …………………………………..
- Patient Room #: ………………………………..

- Forecast Date of discharge: _ _ /_ _ _ / _ _ _ _ (dd/Mmm/yyyy)
- Investigator Name: Last _________________ First _______________

Completion progress of the study forms:

Forms	Paper CRF	OpenClinica electronic CRF
CRF 1: Preoperative Data (Before anaesthesia)		
CRF 2: Intraoperative Data (During anaesthesia and 60 minutes afterwards)		
CRF 3: Perioperative Complications (During anaesthesia and up to 60 minutes afterwards)		
CRF 4: Postanaesthetic Data (Recovery room/Intermediate Care/Intensive Care)		

APRICOT Case Report Form

CRF 1: Preoperative Data
Before anaesthesia

**PATIENT INFORMATION & CONSENT**			
1	**Study Subject ID:** *Enter Study Subject ID in this format xxx-xxx-xxx 3 digit code for the country, 3 digit code for the hospital and 3 digit individual patient number, separated with hyphens.*		|__|__|__|-|__|__|__|-|__|__|__|
2	**Informed consent applicable?** *(choose no if waived by local EC)* ☐ **Yes** ☐ **No**		*2.1 If yes, was consent obtained?* ☐ **Yes** ☐ **No** *2.1.1 If obtained, enter date of Informed Consent in this format dd-Mmm-YYYY (Month in English starting with capital letter)* |__|__|- |__|__|__| -|__|__|__|__|
**I. DEMOGRAPHICS**			
1	**Was the child premature?** *(less than 37 weeks)*	☐ **Yes** ☐ **No** ☐ **Unknown**	*1.1 If yes, gestational age at birth?* **|__|__| weeks** [24-36]
2	**What is the child's age?**	**|__|__| years** [0-15] **|__|__| months** [0-11] **|__|__| days** [0-30]	
3	**Gender:**	☐ **Male** ☐ **Female**	
4	**Ethnicity:**	*(choose single most appropriate)* ☐ **White** ☐ **Spanish/Hispanic/Latino** ☐ **Asian** (e.g. Indian, Pakistani, Bangladeshi, Chinese, Vietnam, etc.) ☐ **Black** (Caribbean, African) ☐ **Arabic** (North Africa, Middle East) ☐ **Other**	*4.1 If other, specify:*
5	**ASA score:**	☐ **I** ☐ **II** ☐ **III** ☐ **IV** ☐ **V**	
6	**Height available?**	☐ **Yes** ☐ **No**	*6.1* **|__|__|__| cm** [40-200]
7	**Weight available?**	☐ **Yes** ☐ **No**	*7.1* **|__|__|__| . |__| kg** [0.0-120.0]

II. MEDICAL HISTORY			
1	**Flu/cold:** *Does child currently (or in the 2 weeks preceding procedure) have flu or a cold?*	☐ Yes ☐ No ☐ Not asked/Not relevant	
2	**Wheezing/whistling:** *Has the child had wheezing or whistling spontaneously or after exercise in the last 12 months?*	☐ Yes ☐ No ☐ Not asked/Not relevant	
3	**Asthma:** *Has the child ever had diagnosed asthma?*	☐ Yes ☐ No ☐ Not asked/Not relevant	
4	**Atopy**: *Is child atopic? (Sneezing, or a runny nose or itchy watery eyes or nose or itchy rash or eczema, in the last 12 months)?*	☐ Yes ☐ No ☐ Not asked/Not relevant	
5	**Allergy:** *Has the child ever had allergy?*	☐ Yes<br>☐ No<br>☐ Not asked/Not relevant	*5.1 If yes, indicate all allergies that apply:*<br>☐ Food ☐ Nut<br>☐ Latex ☐ Antibiotics<br>☐ Other<br>*5.1.1 If other, specify:*
6	**Snoring:** *While sleeping, does the child snore?*	☐ Yes ☐ No ☐ Not asked/Not relevant	
7	**Fever:** *Has the child had a fever in the last 24 hours (+38.5°C)?*	☐ Yes ☐ No ☐ Not asked/Not relevant	
8	**Smoking:** *Does anyone in the family/giving care to the child smoke + than 10 cigarettes/day?*	☐ Yes ☐ No ☐ Not asked/Not relevant	
9	**Anaesthetic complication:** *Has the child had any previous documented anaesthetic complication?*	☐ Yes ☐ No ☐ Not asked/Not relevant	
10	**Medication:** *Does the child take any regular medication, natural products and/or homeopathic products?*	☐ Yes<br>☐ No<br>☐ Not asked/Not relevant	*10.1 If yes, specify:*
11	**Handicap:** *Does the child have metabolic/genetic disorder or neurological impairment?*	☐ Yes ☐ No ☐ Not asked/Not relevant	

III. INDICATION

1	**Type of procedure:**<br>☐ **Surgical**<br>☐ **Non- Surgical procedure**	**1.1 If surgical, indicate type of surgical procedure:**<br>*(tick all that apply)*<br>☐ **Orthopaedic**<br>☐ **Gastro/Abdominal**<br>☐ **Hepato-biliary/Pancreas**<br>☐ **Urological/Kidney**<br>☐ **Ear-Nose-Throat**<br>☐ **Ophthalmology**<br>☐ **Cutaneous/Dermatology**<br>☐ **Plastics (cleft)**<br>☐ **Neurosurgery**<br>☐ **Cardiac surgery**<br>☐ **Thoracic**<br>☐ **Trauma**<br>☐ **Head and Neck**<br>☐ **Other**<br>*1.1.1 If other, specify:*
		**1.2 If non-surgical, indicate Non-Surgical painful procedure:**<br>*(tick all that apply)*<br>☐ **Gastroenterology**<br>☐ **Bronchoscopy**<br>☐ **Dental**<br>☐ **Biopsy**<br>☐ **Bone Marrow & Lumbar puncture**<br>☐ **Ophthalmologic examination**<br>☐ **CT-Scan**<br>☐ **MRI (Magnetic rad. Imaging)**<br>☐ **Venous access**<br>☐ **Burns dressing**<br>☐ **Other non-surgical**<br>*1.2.1 If other, specify:*
2	**Degree of urgency:**	*(choose single most appropriate)*<br>☐ **Elective** ☐ **Urgent** ☐ **Emergency**
3	**Patient type:**	*(choose single most appropriate)*<br>☐ **Outpatient** ☐ **Inpatient**
4	**Procedure Scheduled time:**	*(choose single most appropriate)*<br>☐ **Opening hours of OR** ☐ **After-hours or weekend**
5	**Date Anaesthesia (induction):**<br>*dd/Mmm/yyyy*	**|__|__|- |__|__|__| - |__|__|__|__|**<br>[>=01-Apr-2014]
6	**Time Anaesthesia (induction):**	**|__|__| hrs** [0-23] **|__|__| min** [0-59]

IV. ANAESTHESIA PLAN			
1	**Consultation:** *Was the child seen in a face to face consultation at least 24 hours prior to procedure?*	☐ **Yes** ☐ **No**	
2	**Anaesthesia team in charge:** *What kind of Anaesthesiologist?*	*(tick all that apply)*<br>☐ **Specialist anaesthesiologist with mainly paediatric practice (>80%)**<br>☐ **Specialist anaesthesiologist with frequent paediatric anaesthesia cases (50-80%)**<br>☐ **Specialist anaesthesiologist with occasional paediatric anaesthesia cases (<50%)**<br>☐ **Anaesthesiologist in training**<br>☐ **Anaesthetic nurse**<br>☐ **Anaesthetic technician**	
3	**Experience:** *For how many years the senior person in charge of the patient has been practicing?*	**|__|__| yrs** [1-50]	
4	**Pre-Medications:** *Was there any medication taken by the child just before the anaesthesia?*	☐ **Yes** ☐ **No**	**4.1 If yes, indicate medication(s):** *(tick all that apply)*<br>☐ **Midazolam oral**<br>☐ **Midazolam rectal**<br>☐ **Clonidine**<br>☐ **Paracetamol oral**<br>☐ **Paracetamol rectal**<br>☐ **Local Anaesthetic Cream**<br>☐ **Other**<br>*4.1.1 If other, specify:*
5	**Parental presence:** *Was the child accompanied by a parent during the induction?*	☐ **Yes** ☐ **No**	
6	**Monitoring:** *Specify*	*(choose single most appropriate)*<br>☐ **Standard: ECG, SpO_2 anaesthetic agent, capnography, NIBP, temp**<br>☐ **Standard + : Arterial, central line**<br>☐ **Standard ++: NIRS, EEG derivated data**<br>☐ **Standard minus: one of the equipment missing**	

CRF 2 : Intraoperative Data<br>*During anaesthesia and 60 minutes afterwards*			
1	**Type of Anaesthesia**	*(choose single most appropriate)*<br>☐ Sedation<br>☐ General Anaesthesia<br>☐ None	
	**I. SEDATION**		
1.1	**If Sedation, indicate Sedation Drug**<br>*then please complete section III Regional Anaesthesia*	*(tick all that apply)*<br>☐ Propofol<br>☐ Ketamine<br>☐ Midazolam<br>☐ Dexmedetomidine<br>☐ OPIATE<br>☐ Other	**1.1.1 If opiate(s), specify:**<br>*(tick all that apply)*<br>☐ Fentanyl<br>☐ Alfentanil<br>☐ Remifentanil<br>☐ Morphine<br>☐ Other
	**II. GENERAL ANAESTHESIA**		
1	**Induction type at onset:**	*(tick all that apply)*<br>☐ Inhalational ☐ Intravenous<br>☐ Intramuscular	
2	**Rapid Sequence Induction:**	*(choose single most appropriate)*<br>☐ No mask ventilation<br>☐ Modified with mask ventilation<br>☐ No	
3	**Cricoid pressure:**	☐ Yes ☐ No	
4	**Induction Drug:**	*(tick all that apply)*<br>☐ Sevoflurane<br>☐ Halothane<br>☐ Propofol<br>☐ Thiopentone<br>☐ Etomidate<br>☐ Ketamine<br>☐ Midazolam<br>☐ Atropine<br>☐ OPIATE	**4.1 If opiate(s), specify:**<br>*(tick all that apply)*<br>☐ Sufentanil<br>☐ Fentanyl<br>☐ Alfentanil<br>☐ Remifentanil<br>☐ Morphine<br>☐ Other
5	**Nitrous Oxide at induction (N_2O):**	☐ Yes ☐ No	
6	**Neuromuscular blocking agent(s):**	*(tick all that apply)*<br>☐ None<br>☐ Succinylcholine<br>☐ Cisatracurium<br>☐ Atracurium<br>☐ Rocuronium<br>☐ Vecuronium<br>☐ Other<br>☐ Unknown	**6.1 If neuromuscular blocking agent(s) used, neuromuscular monitoring?**<br>☐ Yes ☐ No<br>**6.2 If neuromuscular blocking agent used, reversal at the end?**<br>*(choose most appropriate)*<br>☐ Neostigmine<br>☐ Sugammadex<br>☐ No

7	**Maintenance: inhalational, TIVA**	*(tick all that apply)*<br>☐ **Sevoflurane**<br>☐ **Isoflurane**<br>☐ **Desflurane**<br>☐ **Propofol**<br>☐ **Ketamine**<br>☐ **OPIATE**<br>☐ **Other**	**7.1 If opiate, specify:** *(tick all that apply)*<br>☐ **Sufentanil**<br>☐ **Fentanyl**<br>☐ **Alfentanil**<br>☐ **Remifentanil**<br>☐ **Morphine**<br>☐ **Other**
8	**Carrier gas:**	*(choose single most appropriate)*<br>☐ **Oxygen**<br>☐ **Oxygen + air**	☐ **Oxygen + N_2O**<br>☐ **Air**

III. REGIONAL ANAESTHESIA

1	**Regional Anaesthesia:**	*(choose single most appropriate)*<br>☐ **Alone**<br>☐ **With IV sedation**<br>☐ **With GA**<br>☐ **None**	
1.1	If regional Anaesthesia, specify Type:	*(choose single most appropriate)*<br>☐ **Nerve stimulation (NS)**<br>☐ **Ultrasound (US)**<br>☐ **Combination of both NS and US**<br>☐ **None of the above (landmarks)**	
1.2	If regional Anaesthesia, specify Neuraxial block:	*(tick all that apply)*<br>☐ **Spinal**<br>☐ **Caudal**<br>☐ **Lumbar epidural**<br>☐ **Thoracic epidural**<br>☐ **Upper limb**<br>☐ **Lower limb**<br>☐ **Ilioinguinal**<br>☐ **TAP**<br>☐ **Intercostal**<br>☐ **Paraumbilical**<br>☐ **Penile**<br>☐ **Pudendal**<br>☐ **Craniofacial**<br>☐ **Infiltration of the wound**<br>☐ **Other**	
1.3	If regional Anaesthesia test dose:	*(choose single most appropriate)*<br>☐ **No**<br>☐ **Local Anaesthetic with no epinephrine**<br>☐ **Local Anaesthetic with epinephrine**	
1.4	If regional Anesthesia, specify Drugs:	*(tick all that apply)*<br>☐ **Bupivacaine**<br>☐ **Levobupivacaine**<br>☐ **Ropivacaine**<br>☐ **Lidocaine**<br>☐ **Prilocaine**<br>☐ **Epinephrine**<br>☐ **Clonidine**<br>☐ **Other**	*1.4.1 If other, specify:*

IV. AIRWAY MANAGEMENT

1		**Specify type of interface for airway management:** *(choose single most appropriate)* ☐ **Face mask** ☐ **SGAW (Supraglottic airway)** ☐ **ETT (Endotracheal tube)** ☐ **Other** ☐ **None**	*1.1 If other, specify:*
**If SGAW or ETT =>**	2	Insertion at: *(choose single most appropriate)* ☐ **First attempt** ☐ **Second attempt** ☐ **More than 3 attempts** ☐ **Unsucessfull**	
=>	3	Change to other anaesthesiologist in charge? ☐ **Yes** ☐ **No**	
**If SGAW =>**	4	Indicate type: *(choose single most appropriate)* ☐ **Classic** ☐ **ProSeal** ☐ **Reinforced/flexible LMA** ☐ **ILMA** ☐ **IGel** ☐ **Other**	*4.1 If other, specify*
=>	5	Indicate removal details: ☐ **Awake** ☐ **Deep anaesthesia**	
**If ETT =>**	4	Indicate Tube Type: ☐ **Cuffed** ☐ **Uncuffed**	***4.1 If cuffed, specify: Monitoring cuff pressure?*** ☐ **Yes** ☐ **No** ***4.2 If uncuffed, specify: Packing?*** ☐ **Yes** ☐ **No**
=>	5	Use of guide for intubation? ☐ **Stylet** ☐ **Bougie** ☐ **None**	
	6	Intubation: *(choose single most appropriate)* ☐ **Direct laryngoscopy** ☐ **Fiberoptic** ☐ **ILMA** ☐ **Other** ☐ **Videoendoscopy**	*6.1 If other, specify:*
=>	7	Intubation way: ☐ **Oral** ☐ **Nasal**	
=>	8	Tube Type: *(choose single most appropriate)* ☐ **Classic** ☐ **Nasal preformed** ☐ **RAE** ☐ **Other** ☐ **Reinforced tube**	*8.1 If other, specify:*
=>	9	Vocal Cords sprayed with lignocaine prior to intubation? ☐ **Yes** ☐ **No**	
=>	10	Cormack-Lehane score: *(choose single most appropriate)* ☐ **1** ☐ **2** ☐ **3** ☐ **4**	
=>	11	Removal of tube: ☐ **Awake** ☐ **Deep** ☐ **Left intubated**	

V. VENTILATION

1	**Ventilation type:**<br>*(choose single most appropriate)*<br>☐ Spontaneous ventilation<br>☐ Pressure support ventilation<br>☐ Mechanical ventilation	***1.1 If mechanical ventilation specify:***<br>*(choose single most appropriate)*<br>☐ Volume controlled<br>☐ Pressure controlled<br>☐ Pressure regulated volume controlled<br>☐ Jet ☐ Other	*1.1.1 If other, specify:*
2	**Is the child less than 6 months?**<br>☐ Yes ☐ No	***2.1 If yes, mild episode of mild Hypoxaemia for at least 2 min. during anaesthesia management? (86% < SaO2 < 90%):*** ☐ Yes ☐ No	
		2.2 If yes, severe episode of severe Hypoxaemia for at least 2 min. during anaesthesia management? (SaO2 < 85%): ☐ Yes ☐ No	
		2.3 If yes, <u>lowest</u> etCO2 < 4.5 **kPa or < 35 mmHg** ***for at least 2 min. during anaesthesia management?***<br>☐ Yes ☐ No	

VI. FLUIDS

Has patient received intraoperative fluids? ☐ Yes ☐ No **If yes complete this section**

1	**Glucose-containing fluid?**<br>☐ Yes ☐ No	***1.1 If yes, provide concentration***<br>☐ 1% ☐ 2%<br>☐ 2.5% ☐ 5%<br>☐ 10% ☐ other %	
2	**Crystalloids administered:** *(tick all that apply)*<br>☐ No ☐ Normal Saline<br>☐ Ringer Lactate ☐ Ringer acetate<br>☐ Hartmann solution ☐ Other	*2.1 If other, specify:*	
3	**Colloids administered:** *(choose single most appropriate)*<br>☐ No ☐ Synthetic colloids<br>☐ Albumin ☐ Other	*3.1 If other, specify:*	
4	**Blood products?**<br>☐ Yes ☐ No	***4.1 Packed RBCs:***<br>☐ Yes ☐ No	***4.1.1 # of packs RBC***<br>**|__|__| packs** [1-99]
		4.2 FFP:<br>☐ Yes ☐ No	***4.2.1 # of packs FFP***<br>**|__|__| packs** [1-99]
		4.3 Platelets:<br>☐ Yes ☐ No	
		4.4 Fibrinogen:<br>☐ Yes ☐ No	

VII. TIMING

1	**Duration of surgical/non-surgical procedure**	**|__|__|__| min** [1-999]
2	**Duration of anaesthesia**<br>*(wheel-in, wheel-out of operation room)*	**|__|__|__| min** [1-999]

CRF 3: Perioperative Complications<br>During anaesthesia and up to 60 minutes afterwards<br>*For definitions of Complications please refer to last page of CRF or Appendix 5 of protocol*		
**Has patient had any perioperative complications? ☐ Yes ☐ No** (if yes complete this section)		
1	**Bronchospasm?**	☐ **Yes** ☐ **No**
**1.1**	Bronchospasm, Time of occurrence? *(tick all that apply)*<br>☐ **Induction** ☐ **Maintenance** ☐ **Awakening** ☐ **PACU**	
**1.2**	Bronchospasm, specify Treatment:<br>*(tick all that apply)*<br>☐ **Bronchodilator**<br>☐ **Intubation**<br>☐ **Epinephrine**<br>☐ **Other**	*1.2.1 If other, specify:*
**1.3**	Bronchospasm, Outcome of event?<br>*(tick all that apply)*<br>☐ **Uneventful**<br>☐ **Hypoxemia (< 90%)**<br>☐ **Cardiac arrest**<br>☐ **Prolonged intubation** ☐ **Other**	*1.3.1 If other, specify:*
2	**Laryngospasm?**	☐ **Yes** ☐ **No**
**2.1**	Laryngospasm, Time of occurrence? *(tick all that apply)*<br>☐ **Induction** ☐ **Maintenance** ☐ **Awakening** ☐ **PACU**	
**2.2**	Laryngospasm, specify Treatment:<br>*(tick all that apply)*<br>☐ **Propofol**<br>☐ **Succinycholine**<br>☐ **Intubation**<br>☐ **Other**	*2.2.1 If other, specify*
**2.3**	Laryngospasm, Outcome of event?<br>*(tick all that apply)*<br>☐ **Uneventful** ☐ **Pulmonary edema** ☐ **Cardiac arrest** ☐ **Prolonged intubation**	
3	**Pulmonary aspiration?**	☐ **Yes** ☐ **No**
**3.1**	Pulmonary Aspiration, Time of occurrence? *(tick all that apply)*<br>☐ **Induction** ☐ **Maintenance** ☐ **Awakening** ☐ **PACU**	
**3.2**	Pulmonary Aspiration, specify<br>Treatment: *(tick all that apply)*<br>☐ **Broncho-tracheal suction**<br>☐ **Intubation**<br>☐ **CPAP**<br>☐ **Bronchodilator** ☐ **Other**	*3.2.1 If other, specify:*
**3.3**	Pulmonary Aspiration, Outcome of event? *(tick all that apply)*<br>☐ **Uneventful** ☐ **Hypoxemia**<br>☐ **Cardiac arrest** ☐ **Other**<br>☐ **Prolonged intubation**	*3.3.1 If other, specify:*

4	**Drug error?**	☐ **Yes** ☐ **No**
4.1	Drug error, Time of occurrence? *(tick all that apply)*<br>☐ **Induction** ☐ **Maintenance** ☐ **Awakening** ☐ **PACU**	
4.2	Drug error, Type? *(tick all that apply)*<br>☐ **Wrong dosage** ☐ **Wrong product** ☐ **Wrong site of administration**	
4.3	Drug error, Treatment necessary?<br>☐ **Yes** ☐ **No**	*4.3.1 Specify treatment:*
4.4	Drug error, Outcome of event? *(tick all that apply)*<br>☐ **Minor sequellae**<br>☐ **Major sequellae**<br>☐ **Cardiac arrest** ☐ **Other**	*4.4.1 If other, specify:*
5	**Anaphylaxis?**	☐ **Yes** ☐ **No**
5.1	Anaphylaxis, Time of occurrence? *(tick all that apply)*<br>☐ **Induction** ☐ **Maintenance** ☐ **Awakening** ☐ **PACU**	
5.2	Anaphylaxis, specify Treatment: *(tick all that apply)*<br>☐ **Fluid resuscitation** ☐ **First Intubation**<br>☐ **Epinephrine** ☐ **Other**<br>☐ **Bronchodilator**	*5.2.1 If other, specify:*
5.3	Anaphylaxis, specify Outcome of event? *(tick all that apply)*<br>☐ **Uneventful** ☐ **Pulmonary edema** ☐ **Cardiac arrest** ☐ **Prolonged intubation**	
6	**Cardiovascular instability/Hemodynamic?**	☐ **Yes** ☐ **No**
6.1	Cardiovascular Instability, Time of occurrence? *(tick all that apply)*<br>☐ **Induction** ☐ **Maintenance** ☐ **Awakening** ☐ **PACU**	
6.2	Cardiovascular instability, Type?*(tick all that apply)*<br>☐ **Bleeding** ☐ **Arrhythmia**<br>☐ **Hypotension** ☐ **Vasodilatation**<br>☐ **Other**	*6.2.1 If other, specify:*
6.3	Cardiovascular instability, specify Treatment:<br>*(tick all that apply)*<br>☐ **Fluid resuscitation (non blood)**<br>☐ **Blood product**<br>☐ **Vasopressor**<br>☐ **Atropine**<br>☐ **Defibrillation** ☐ **Other**	*6.3.1 If other, specify:*
6.4	If yes Cardiovascular instability, specify Outcome of event? *(tick all that apply)*<br>☐ **Uneventful**<br>☐ **Coagulopathy**<br>☐ **Cardiac arrest** ☐ **Other**	*6.4.1. If other, specify:*

**7**	**Neurological Damage(s)?**	☐ **Yes** ☐ **No**	
7.1	If yes Neurological Damage<br>Time of occurrence?<br>*(tick all that apply)*	☐ **Induction**<br>☐ **Maintenance**<br>☐ **Awakening**<br>☐ **PACU**	
7.2	Neurological Damage Treatment necessary?	☐ **Yes** ☐ **No**	*7.2.1 If yes, specify:*
7.3	If yes Neurological Damage,<br>specify Outcome of event?<br>*(tick all that apply)*	☐ **Minor sequellae**<br>☐ **Major sequellae**<br>☐ **Death**<br>☐ **Other**	*7.3.1 If other, specify:*
**8**	**Perianaesthetic Cardiac Arrest?**	☐ **Yes** ☐ **No**	
8.1	If yes Cardiac Arrest<br>Time of occurrence? *(tick all that apply)*	☐ **Induction**<br>☐ **Maintenance**<br>☐ **Awakening**<br>☐ **PACU**	
8.2	If yes Cardiac Arrest Treatment<br>*(tick all that apply)*	☐ **Closed Chest massage**<br>☐ **Open Chest massage**<br>☐ **Defibrillation**<br>☐ **Epinephrine**<br>☐ **Other**	*8.2.1 If other, specify:*
8.3	If yes Cardiac Arrest,<br>specify Outcome of event?<br>*(tick all that apply)*	☐ **Uneventful**<br>☐ **Cardiac failure**<br>☐ **Death**<br>☐ **Other**	*8.3.1 If other, specify:*
**9**	**Postanaesthetic Stridor?**	☐ **Yes** ☐ **No**	
9.1	If yes Stridor, Time of occurrence?<br>*(tick all that apply)*	☐ **Awakening**<br>☐ **PACU**	
9.2	Stridor, specify Treatment: *(tick all that apply)*	☐ **CPAP**<br>☐ **Epinephrine**<br>☐ **Other**	*9.2.1 If other, specify:*
9.3	If yes Stridor, specify Outcome of event?<br>*(choose single most appropriate)*	☐ **Uneventful**<br>☐ **Intubation**<br>☐ **Other**	*9.3.1 If other, specify:*

CRF 4: Postanaesthetic Data

Recovery room/Intermediate Care/Intensive Care

1	**Where was the patient transferred after the anaesthesia procedure?**	*(choose single most appropriate)*<br>☐ **Ward**<br>☐ **Recovery room**<br>☐ **Intermediate Care**<br>☐ **Intensive Care**	
2	**Training of the person taking care of the child (after transfer)?**	*(choose single most appropriate)*<br>☐ **Qualified nurse**<br>☐ **Nurse in training**<br>☐ **Other**	
3	**Oxygen delivery?**	*(choose single most appropriate)*<br>☐ **Yes, systematic**<br>☐ **Yes, if necessary**<br>☐ **No**	
3.1	If yes Oxygen delivery, duration?	**|__|__|__| min** [1-999]<br>☐ **Unknown**	
4	**Duration of stay in Recovery room/Intermediate Care/Intensive Care?**	**|__|__|** [0-30] **days |__|__| hours** [0-23] **|__|__| min** [0-59]	
5	**Patient Status at 30 days (or discharge date)**	*(choose single most appropriate)*<br>☐ **Discharged to home**<br>☐ **Discharged to acute centre**<br>☐ **Discharged to Convalescent**<br>☐ **Still in Hospital on day 30**<br>☐ **Dead**	**5.1 Date of discharge or death:**<br>**|__|__| - |__|__|__| -**<br>**|__|__|__|__|**<br>**[>=01-Apr-2014]**<br><br>**5.2 If dead, Suspected cause of death :**

Critical Events: Definitions

**Anaphylaxis:**	is defined by the occurrence of any suspected IgE or non-IgE mediated severe allergic reaction leading to cardiovascular instability and/or severe bronchospasm and requiring immediate resuscitation (fluid resuscitation and epinephrine)[19, 20].
**Bronchospasm:**	is defined as an increased respiratory effort, especially during expiration, and wheeze on auscultation. If the patient is ventilated, bronchospasm may also be considered if a significant increase in peak inspiratory pressure (under volume controlled ventilation) or significant decrease in tidal volume (under pressure controlled ventilation) are observed. In all cases, any episode of airway constriction requiring the administration of a bronchodilator will be recorded.[4]
**Cardiac Arrest: (Perianaesthetic)**	is defined as cessation of circulation (e.g. Pulseless electric activity, asystole, ventricular fibrillation/tachycardia) requiring open or closed chest compressions, or resulting in death, while the patient is in the care of the anaesthetic team. Three advocators, among the local anaesthetic team, will determine the anaesthetic responsibility for cardiac arrest [10, 11].
**Cardiovascular instability:**	is defined by the occurrence of either one of the following:<br>- **cardiac arrhythmia** defined as ECG evidence of cardiac rhythm disturbance considered by clinical staff to be severe enough to require treatment (e.g. anti-arrhythmic agents, vasoactive agents, intravenous fluid, etc.). This includes arrhythmias occurring following regional analgesia and requiring intervention. For example: bradycardia requiring atropine, supraventricular tachycardia, atrial or - - ventricular tachyarrhythmia, torsade de Pointe, etc.<br>- **hypotension** defined as a drop in blood pressure requiring intervention by the anaesthesiologist (fluid resuscitation and/or the administration of vasoactive drugs)<br>- **bleeding** resulting in hypotension and necessitating unanticipated and unpredicted blood transfusion<br>- **cardiovascular instability** despite anticipated bleeding and transfusion (e.g.: liver transplant, scoliosis…)
**Drug error:**	is defined as the administration of a wrong drug, or a wrong dose given by any route, or a wrong site of administration, that has led to either respiratory/cardiac/neurological consequence or to an unplanned admission to the ICU or prolonged hospitalization.
**Laryngospasm:**	is defined either as complete airway obstruction associated with rigidity of the abdominal and chest walls and leading to unsuccessful child's ventilation, or glottic closure associated with chest movement but silent unsuccessful child's respiratory efforts and assisted ventilation, unrelieved in both situations with simple jaw thrust and CPAP manoeuvres and requiring the administration of medication (propofol, suxamethonium etc.) and/or tracheal intubation.[4][16]
**Neurological damage:**	is defined in case of regional anaesthesia by the occurrence of nerve injury or spinal cord insult or seizure requiring resuscitation. In case of general anaesthesia, any episode of seizure, pressures sore, episodes of loss of vision or new onset of central neurological impairment. This includes peripheral nerve injury following positioning (ulnar nerve, external popliteal nerve) or puncture (median or ulnar nerve).
**Pulmonary Aspiration:**	is defined as the presence of any non-respiratory secretions (bilious or particulate) in the airway as evidenced by laryngoscopy, suctioning, or bronchoscopy. In a situation where there was suspicion of pulmonary aspiration but no positive aspiration of non-respiratory secretions, new clinical and/or chest X-ray signs consistent with aspiration are accepted as evidence for it (e.g., new wheeze or crackles in the chest after regurgitation or vomiting incident). [17, 18]
**Stridor (Postanaesthetic):**	is defined as a severe inspiratory flow limitation with sternal retraction, intrathoracic pressure swing, and potentially cyanosis occurring in the PACU and necessitating the administration of oxygen, intravenous steroids and/or epinephrine (nebulisation) or tracheal intubation

Other: Definitions

**Sedation:**	**Minimal sedation:** Also known as anxiolysis. A drug-induced state during which the patient responds normally to verbal commands. Cognitive function and coordination may be impaired. Ventilatory and cardiovascular functions are unaffected.<br><br>**Moderate sedation/analgesia (conscious sedation):** A drug-induced depression of consciousness during which the patient responds purposefully to verbal command, either alone or accompanied by light tactile stimulation. No interventions are necessary to maintain a patent airway. Spontaneous ventilation is adequate. Cardiovascular function is usually maintained.<br><br>**Deep sedation/analgesia:** A drug-induced depression of consciousness during which the patient cannot be easily aroused, but responds purposefully* following repeated or painful stimulation. Independent ventilatory function may be impaired. The patient may require assistance to maintain a patent airway. Spontaneous ventilation may be inadequate. Cardiovascular function is usually maintained.
**General anesthesia:**	A drug-induced loss of consciousness during which the patient is not arousable, even to painful stimuli. The ability to maintain independent ventilatory function is often impaired. Assistance is often required in maintaining a patent airway. Positive pressure ventilation may be required due to depressed spontaneous ventilation or drug-induced depression of neuromuscular function. Cardiovascular function may be impaired.<br><br>** Reflex withdrawal from a painful stimulus is NOT considered a purposeful response*

NECTARINE: NE*onate* – C*hildren* S*T**udy of* A*naesthesia p*R*actice* IN E*urope*

NECTARINE Screening – Inclusion Form

Inclusion criteria
***Corrected age calculation**<br><br>A. Baby's age at birth since last mother's menses (gestational age) |__|__| weeks<br>B. Baby's age: number of weeks since birth |__|__| weeks<br><br>**Corrected age = value of A plus value of B =** |__|__| weeks<br><br>**Is Corrected age ≤ 60 weeks ☐ Yes ☐ No**
If "YES", INCLUDE patient and enter in the study => Paper CRF and electronic CRF should be completed.<br>If "NO", DO NOT INCLUDE patient in the study => Paper CRF and electronic CRF should NOT be completed.

NECTARINE: NEonate – Children sTudy of Anaesthesia pRactice IN Europe

NECTARINE Patient Confidential Identification CRF Coversheet

This coversheet intends to help site staff and local investigator linking local patient data to the study specific study patient code and this sheet is used to facilitate the task to the investigators, and can be filled to your convenience. After completing follow-up, this coversheet should be saved apart from the CRF and filed separately in a secure place. The information on this coversheet will NOT be collected in the CRF. It is for local use ONLY.

IDENTIFYING DATA			
A	**Date paper CRF created**	|__|__| / |__|__|__| / |__|__|__|__| (dd/Mmm/YYYY)	
B	**Date of birth**	|__|__| / |__|__|__| / |__|__|__|__| (dd/Mmm/YYYY)	
C1	**Patient Code (OpenClinica eCRF ID number)**	|__|__|__|-|__|__|__|-|__|__|__| (*xxx-xxx-xxx 3 digit code for the country, 3 digit code for the hospital and 3 digit individual patient number)*	
C2	**Identification** (fill in with available data – only for local follow-up use)**:**	Patient Hospital/local Identification Number (handwritten or sticker):<br>Child's First name:<br>Child's Last name:	
		**Family Address**:	
		**Parent**<br>Name:<br>Phone:<br>Email:	**Phone number** to be used for the 90 days Follow up:
		**Family doctor**<br>Name:<br>Phone:<br>email:	

**Record here multiple anaesthesia dates (if applicable):**		1 |__|__|/|__|__|__|/|__|__|__|__|	2 |__|__| / |__|__|__|/|__|__|__|__|
3|__|__|/|__|__|__|/|__|__|__|__|	4|__|__|/|__|__|__|/|__|__|__|__|	5 |__|__|/|__|__|__|/|__|__|__|__|	6 |__|__| / |__|__|__| /|__|__|__|__|
7|__|__|/|__|__|__|/|__|__|__|__|	8|__|__|/|__|__|__|/|__|__|__|__|	9|__|__|/|__|__|__|/|__|__|__|__|	10|__|__| / |__|__|__|/|__|__|__|__|

**Completion progress of the study forms:**	**Paper CRF**	**OpenClinica electronic CRF**
**CRF1: (complete 1x)**<br>- **Patient Data & Medical History**	☐	☐
**CRF2: (repeat for each anaesthesia)**<br>- **Anaesthesia Data Intraoperative Data**<br>- **Interventions during anaesthesia**<br>- **Recovery Room**	☐<br>☐<br>☐	☐<br>☐<br>☐
**CRF3: (complete 1x after last anaesthesia)**<br>- **CRF3-A: Follow Up at 30 days**<br>- **CRF3-B: Follow Up at 90 days**	☐<br>☐	☐<br>☐

Appendix 2 - CRF

NECTARINE: NE*onate* – C*hildren* S*T**udy of* A*naesthesia* p*R**actice* IN E*urope*

NECTARINE Case Report Form

CRF 1: Preoperative (Non-Repetitive = 1 per patient)			
		**PATIENT DATA**	
**1**	**Study Subject ID:**	|__|__|__|-|__|__|__|-|__|__|__| *Enter Study Subject ID in this format xxx-xxx-xxx 3 digit code for the country, 3 digit code for the hospital and 3 digit individual patient number*	
**2**	**Informed consent applicable?**<br>*(choose no if local authorities/ethics gave written exemption of the consent process)*<br>☐ No ☐ Yes	2.1 *If yes, was consent obtained?*<br>☐ No ☐ Yes<br>*2.1.1 If obtained, enter date of Informed Consent in this format dd-Mmm-YYYY (Month in English starting with capital letter)*<br>|__|__|- |__|__|__| -|__|__|__|__|	
**3**	**Gestational age at birth**	**|__|__|** weeks [22-40]	
**4**	**Birth weight:**	**|__|.|__|__||__|** kg [0.000-9.999]	
**5**	**Gender:**	☐ Male ☐ Female	
**6**	**Maternal medications and health issues during pregnancy:**	☐ No<br>☐ Yes<br>☐ Info not available	6.1 *If yes (tick all that apply):*<br>☐ NSAID, Aspirin<br>☐ Anti-hypertensive drugs<br>☐ Alcohol/opiates/narcotics<br>☐ Preeclampsia / Eclampsia<br>☐ If other, not above reported, tick here
**7**	**Mode of delivery:**	*tick single most appropriate*:<br>☐ Vaginal ☐ Caesarean Section ☐ Info not available	
**8**<br>**9**	**APGAR score:**	1 minute: **|__|__|** [00-10] ☐ info not Available<br>5 minutes: **|__|__|** [00-10] ☐ info not Available	
**10**	**Has the child any known congenital abnormality?**	☐ No ☐ Yes	10.1 *If yes, tick all that apply (do not ask the parents for this. It should be filled from the medical record):*<br>☐ Known or suspected myopathy<br>☐ Congenital heart disease<br>☐ Known or suspected metabolic disorder<br>☐ Chromosomopathy<br>☐ Other (non-cardiac) congenital malformations

NECTARINE

European Society of Anaesthesiology ESA

CRF2: ANAESTHESIA DATA (Repeat CRF2 for each anaesthesia)

I. MEDICAL HISTORY

1	**History of apnoea or respiratory support?**	☐ No ☐ Yes	1.1 *If yes, specify (tick all that apply):*<br>☐ Methyl-xanthine (caffeine)<br>☐ ETT ☐ CPAP ☐ O_2
2	**History of Intra-Ventricular-Haemorrhage:**	☐ No ☐ Yes	2.1 *If yes, provide grade:*<br>|__| [0-4] ☐ Grade not available
3	**History of ECMO support:**	☐ No ☐ Yes	
4	**History of patent ductus arteriosus (PDA):**	☐ No ☐ Yes	4.1 *If yes, was treated? (tick all that apply):*<br>☐ No treatment<br>☐ Treated surgically<br>☐ Treated medically
5	**History of previous surgery?**	☐ **No**<br>☐ **Yes**	5.1 *If yes, specify number of previous surgeries:*<br>|__|__| [1-10]<br>5.2 *If yes, is current surgery due to a complication/incomplete/re-do previous surgery:* ☐ No ☐ Yes

II. CURRENT MEDICAL CONDITION (THE DAY OF ANAESTHESIA)

1	**Study Subject ID:**<br>|__|__|__|-|__|__|__|__|-|__|__|__|__|	2	**Subject multiple anaesthesia #**<br>*Number of this anaesthesia within NECTARINE:* |__|__| [01-10]
3	**Baby's age on day of anaesthesia:**<br>|__|__| weeks [0-40] & |__| days [0-6]	4	**Weight on day of anaesthesia:**<br>|__|.|__|__|__| kg [0.000-9.999]
5	**Where was the child admitted from?**<br>☐ Admitted from home<br>☐ Admitted from ward<br>☐ Admitted from another hospital<br>☐ Admitted from ICU	6	**Child breathing condition the day of anaesthesia:**<br>☐ No oxygen, no ventilation assistance<br>☐ Patient under spontaneous ventilation with O_2<br>☐ Patient under NIV with CPAP<br>☐ Patient INTUBATED on conventional ventilation<br>☐ Patient INTUBATED on HFOV<br>☐ Patient on ECMO<br>6.1 *If patient already intubated, what is the main reason for ETT:*<br>☐ Clinically unstable ☐ For surgery ☐ Other
7	**Place where procedure is performed:**<br>*(choose single)*<br>☐ Operating Room/diagnostic suite<br>☐ ICU	8	**Is the child on one or more than the follow medications?**<br>*(tick all that apply)*<br>☐ Benzodiazepines<br>☐ Opioids<br>☐ Dexmedetomidine/Clonidine<br>☐ Muscle relaxant<br>☐ Ketamine<br>☐ Vasopressors/Inotropes (e.g.noradrenaline, dopamine, etc.)<br>☐ Diuretics<br>☐ Other than the above reported medications<br>☐ No medication

III. ASSESSMENT OF THE CHILD AT THE TIME OF ANAESTHESIA

1	**Current RESPIRATORY and airway problems:**<br>☐ No ☐ Yes ☐ info not available<br>1.1 *If yes, (tick all that apply):*<br>☐ Broncho-pulmonary dysplasia<br>☐ Laryngo-tracheomalacia<br>☐ Stridor<br>☐ Pneumonia<br>☐ Other<br>1.1.1 *If other, specify:*	2	**Current CARDIOVASCULAR problems:**<br>☐ No ☐ Yes ☐ info not available<br>2.1 *If yes, (tick all that apply):*<br>☐ Cardiac murmur of any kind<br>☐ Patent Ductus Arteriosus<br>☐ Pulmonary Hypertension (ultrasound or catheter diagnosis)<br>☐ Cyanosis (Right to Left shunt)<br>☐ Other<br>2.1.1 *If other, specify:*
3	**Current METABOLIC problems:**<br>☐ No ☐ Yes ☐ info not available<br>3.1 *If yes, (tick all that apply):*<br>☐ Sepsis<br>☐ Jaundice (Increased Bilirubin)<br>☐ Metabolic alkalosis MB_PRB_Y If yes, tick all that apply:<br>☐ Metabolic acidosis<br>☐ Other<br>3.1.1 *If other, specify:*	4	**Current neurologic/BRAIN problems:**<br>☐ No ☐ Yes ☐ info not available<br>4.1 *If yes, (tick all that apply):*<br>☐ Hydrocephalus (with or without ventricular shunt)<br>☐ Seizures<br>☐ Periventricular leucomalacia<br>☐ Retinopathy of prematurity<br>☐ Other<br>4.1.1 *If other, specify:*
5	**Current RENAL problems:**<br>☐ No ☐ Yes ☐ info not available<br>5.1 *If yes, (tick all that apply):*<br>☐ Renal insufficiency<br>☐ Renal dysplasia<br>☐ Other<br>*5.1.1 If other, specify:*	6	**ASA score** *(tick single most appropriate)*<br>☐ I *(only applicable if > 3 months)*<br>☐ II<br>☐ III<br>☐ IV<br>☐ V

IV. BASELINE PHYSIOLOGICAL & METABOLIC PARAMETERS

Physiological parameters: *fill all available items based on what you consider as baseline physiological parameters*

1	**Specify when baseline parameters were taken?** *(tick single most appropriate)*	☐ Medical record (within the 24 hrs)<br>☐ Before Induction<br>☐ After induction, first measurement	
1.1	**Systolic blood pressure**	|__|__|__| mmHg [0-199]	☐ Not Available
1.2	**Mean blood pressure**	|__|__|__| mmHg [0-199]	☐ Not Available
1.3	**Diastolic blood pressure**	|__|__|__| mmHg [0-199]	☐ Not Available
1.4	**Heart Rate**	|__|__|__| beats/min. [0-299]	☐ Not Available
1.5	**NIRS**	|__|__|__| [0-100]	☐ Not Available
1.6	**Pre-ductal SpO_2**	|__|__|__| % [0-100]	☐ Not Relevant (only in the neonate)<br>☐ Not Available
1.7	**Post-ductal SpO_2**	|__|__|__| % [0-100]	☐ Not Relevant (only in the neonate)<br>☐ Not Available
1.8	**Arterial/Venous CO_2**	|__|__| mmHg [20-99]<br>☐ Arterial ☐ Venous	☐ Not Available
1.9	**Body temperature**	|__|__|.|__| °C [34.0-40.0]	☐ Not Available

Metabolic parameters:
fill available items for metabolic parameters, referring to preoperative assessment or early intraoperative

2	**Na** |__|__|__|.|__|	*Select unit used*<br>☐ mEq/L [99.9-199.9]<br>☐ mMol/L [99.9-199.9]	☐ Not Available

3	Hb	|__|__|.|__|	*Select unit used*<br>☐ g/dL [0.0-20.0]<br>☐ mMol/L [0.0-20.0]	☐ Not Available
4	Glucose	|__|__|__|	*Select unit used*<br>☐ mg/dL [0.0-999]<br>☐ mMol/L [0.0-50]	☐ Not Available

V. INDICATION

1<br>2	**Date of anaesthesia (induction):**<br>**Time of anaesthesia (induction):**	|__|__|- |__|__|__| - |__|__|__|__| dd/Mmm/yyyy [>=01-Mar-2016]<br>Hours |__|__|[0-23] Minutes|__|__| [0-59]
3<br>4	**Date surgery/procedure started:**<br>**Time surgery/procedure started:**	|__|__|- |__|__|__| - |__|__|__|__| dd/Mmm/yyyy [>=01-Mar-2016]<br>Hours |__|__|[0-23] Minutes|__|__| [0-59]
5	**Degree of urgency:**	*(choose single most appropriate)*<br>☐ Elective ☐ Semi-elective/Urgent ☐ Emergency
6	**Type of procedure:**	☐ Surgical => complete sections 6.1 to 6.10<br>☐ Non-Surgical procedure=> complete section 6.11

If SURGICAL painful procedure *(tick single most relevant; tick main surgery if mixed)***:**

**6.1**	**If surgical, indicate: Minimally invasive surgery? (laparoscopy, thoracoscopy,…)**<br>☐ No<br>☐ Yes	6.1.1 If yes, was it concluded also as minimal?<br>☐ No, converted to open surgery<br>☐ Yes

6.2 Oesophageal, gastro-intestinal surgery:
☐ No ☐ Yes

6.2.1 *If yes, specify*: *(tick most relevant; tick main surgery if mixed)*
☐ Anorectal malformations (PSARP, …)
☐ Biliary atresia: Kasai procedure
☐ Choledochal cyst excision
☐ Diaphragmatic hernia
☐ Fundoplication
☐ Gastrostomy tube
☐ Inguinal Hernia Repair (unilateral or bilateral)
☐ Intestinal obstruction
☐ Liver biopsy
☐ Necrotising Enterocolitis
☐ Oesophageal atresia with or without tracheo-oesophageal fistula
☐ Omphalocoele, gastroschisis
☐ Pyloric stenosis
☐ Ileostomy/Colostomy
☐ Other 6.2.1.1 *If other, specify:*

6.3 Thoracic surgery:
☐ No ☐ Yes

6.3.1 *If yes, specify: (tick single most relevant)*
☐ Congenital lung lesions (Cystic adenomatous malformation)
☐ Lung biopsy
☐ Mediastinal mass
☐ Lobectomy
☐ Other 6.3.1.1 If other, specify:

6.4 Cardiac surgery:
☐ No ☐ Yes

6.4.1 *If yes, specify: (tick single most relevant)*
☐ Aortopexy
☐ Arterial switch operation or other treatment for transposition of great vessels
☐ Blalock shunt
☐ Closure of PDA
☐ Coarctation
☐ Norwood procedure
☐ Pulmonary artery banding
☐ Tetralogy of Fallot
☐ Total abnormal venous return
☐ Other 6.4.1.1 *If other, specify:*

6.5 Genitourinary surgery:

☐ No ☐ Yes

6.5.1 *If yes, specify: (tick single most relevant)*

☐ Peritoneal dialysis catheter
☐ Correction of ureteropelvic junction
☐ Cystostomy
☐ Nephrectomy
☐ Nephroureterectomy, Pyeloplasty
☐ Orchidopexy, Torsion of testis
☐ Ovarian cyst
☐ Circumcision for medical reason
☐ Ritual circumcision
☐ Urethral valves
☐ Other 6.5.1.1 *If other, specify:*

6.6 Neurosurgery:

☐ No ☐ Yes

6.6.1 *If yes, specify: (tick single most relevant)*

☐ Closure of myelomeningocoele
☐ Exploration and decompression spinal canal
☐ Synostosis
☐ Ventricular shunt to abdominal cavity
☐ Ventriculostomy
☐ Other

6.6.1.1 *If other, specify:*

6.7 Ophthalmology surgery:

☐ No ☐ Yes

6.7.1 *If yes, specify: (tick single most relevant)*

☐ Cryotherapy destruction of chorioretinal lesion
☐ Laser destruction of chorioretinal lesion
☐ Phacofragmentation & aspiration of cataract
☐ Probing of nasolacrimal duct
☐ Other

6.7.1.1 If other, specify:

6.8 ENT-Plastic surgery:

☐ No ☐ Yes

6.8.1 *If yes, specify: (tick single most relevant)*

☐ Choanal atresia
☐ Cleft lip
☐ Excision of lesion of external ear
☐ Laser treatment of laryngeal lesions
☐ Lingual frenotomy
☐ Repair and plastic operations on trachea
☐ Other 6.8.1.1 *If other, specify:*

6.9 Orthopaedic surgery:

☐ No ☐ Yes

6.9.1 *If yes, specify: (tick single most relevant)*

☐ Arthrotomy
☐ Clubfoot repair
☐ Excision of soft tissue lesion
☐ Internal fixation of bone
☐ Supernumerary digit (polydactyly)
☐ Other 6.9.1.1 *If other, specify:*

6.10 Dermatology surgery:

☐ No ☐ Yes

6.10.1 *If yes, specify: (tick single most relevant)*

☐ Biopsy of skin and subcutaneous tissue
☐ Excision of skin and subcutaneous tissue
☐ Incision with drainage of skin and subcutaneous tissue (i.e.: anal abscess, etc.)
☐ Operation on skin and subcutaneous tissue
☐ Other 6.10.1.1 *If other, specify:*

If NON-SURGICAL procedure *(tick single most relevant; tick main non-surgery if mixed)*:

6.11

☐ Angiography/embolization
☐ Biopsy
☐ Bronchoscopy
☐ Burns dressing
☐ Cardiac lab (Percutaneous valvuloplasty, rashkind procedure)
☐ CT-Scan
☐ Cystoscopy
☐ Gastroenterology
☐ Infiltration or punction
☐ MRI (Magnetic rad. Imaging)
☐ Ophthalmologic examination/Laser
☐ Pericardial or pleural drainage
☐ PICC line/Central venous/Broviac
☐ Other non-surgica NON_SG_SP If other, specify:

6.11.1 *If other, specify:*

**7**	**Team involved for the anaesthesia management:** *(specify the number of staff members in charge of the patient)*	|__| [0-5] Senior anaesthesiologist (> 5 years from certification)<br>|__| [0-5] Junior anaesthesiologist (< 5 years from certification)<br>|__| [0-5] Anaesthesiologist in training<br>|__| [0-5] Anaesthetic nurse/technician<br>|__| [0-5] ICU personnel (neonatologist or paediatrician)
**8**	**Monitoring:** *(tick all that apply)*	☐ Standard (ECG, SpO_2 , anaesthetic agent, capnography, NIBP, temp)<br>☐ Arterial<br>☐ Central venous line<br>☐ NIRS
**9**	**Anaesthesia Technique:** *(tick single most appropriate)*	☐ General anaesthesia (fill in section V-1)<br>☐ Regional anaesthesia alone (fill in section V-2)<br>☐ Combined general and regional anaesthesia (fill in section V-1 & V-2)

Section V-1. GENERAL ANAESTHESIA

1	**Anaesthesia induction**	*(tick the way of administration of first drug that was administered)* ☐ **Inhalational** ☐ **Intravenous** ☐ **Intramuscular**

Please specify Induction Drug: *(tick all drugs used)*

**1.1**	☐ Sevoflurane	**1.6**	☐ Propofol	**1.11** ☐ Opioid
**1.2**	☐ Halothane	**1.7**	☐ Thiopentone	*If opiate(s), specify:*
**1.3**	☐ Desflurane	**1.8**	☐ Ketamine	1.11.1 ☐ Sufentanil 1.11.4 ☐ Remifentanil
**1.4**	☐ Isoflurane	**1.9**	☐ Atropine	1.11.2 ☐ Fentanyl 1.11.5 ☐ Morphine
**1.5**	☐ Midazolam	**1.10**	☐ Etomidate	1.11.3 ☐ Alfentanil 1.11.6 ☐ Other

**2**	**Neuromuscular blocking agent (NBA) used?** ☐ No ☐ Yes	*2.1 If yes, specify which NBA:* ☐ Succinylcholine ☐ Cisatracurium ☐ Atracurium ☐ Rocuronium ☐ Vecuronium
		2.2 If yes, was NBA given prior or after intubation? ☐ Prior ☐ After
		2.3 If yes, reversal at the end? ☐ Neostigmine ☐ Sugammadex ☐ No
**3**	**Maintenance drugs given?** ☐ No ☐ Yes, specify all drugs for maintenance *(tick all drugs used)*:	

**3.1**	☐ Sevoflurane	**3.6**	☐ Propofol	**3.11** ☐ Opioid
**3.2**	☐ Halothane	**3.7**	☐ Thiopentone	*If opiate(s), specify:*
**3.3**	☐ Desflurane	**3.8**	☐ Ketamine	3.11.1 ☐ Sufentanil 3.11.4 ☐ Remifentanil
**3.4**	☐ Isoflurane	**3.9**	☐ Atropine	3.11.2 ☐ Fentanyl 3.11.5 ☐ Morphine
**3.5**	☐ Midazolam	**3.10**	☐ Etomidate	3.11.3 ☐ Alfentanil 3.11.6 ☐ Other

**4**	**Carrier gas:** *(choose single)* ☐ Oxygen ☐ Oxygen + N_2O ☐ Oxygen + air ☐ Air ☐ Other	
**5**	**Is vasopressor or inotropic** *(i.e. dopamine, norepinephrine, etc.)* **drug infusion part of the anaesthesia management from the beginning?**	☐ No ☐ Yes

Section V-2. REGIONAL ANAESTHESIA

**1**	**Specify block** *(choose single most appropriate)*:	**2**	**Regional catheter for continuous analgesia?**
	☐ Spinal<br>☐ Caudal<br>☐ Lumbar epidural<br>☐ Thoracic epidural<br>☐ Upper limb<br>☐ Lower limb<br>☐ Ilio-inguinal	☐ TAP<br>☐ Intercostal<br>☐ Paraumbilical<br>☐ Penile<br>☐ Craniofacial<br>☐ Infiltration of the wound<br>☐ Other, not above specified	☐ No ☐ Yes

VI. AIRWAY MANAGEMENT

**1**	**Specify type of interface for airway management** *(tick single most appropriate):* ☐ **Face Mask** ☐ **SGAW (Supraglottic Airway)** ☐ **Tracheostomy** ☐ **ETT (Endotracheal tube)** ☐ **Nasal Probe/CPAP/Non-Invasive ventilation** ☐ **None**

**If ETT** =>	**1.1**	Indicate Tube Type: ☐ Cuffed ☐ Uncuffed
=>	**1.2**	Intubation route: ☐ Oral ☐ Nasal
=>	**1.3**	Cormack-Lehane score: *(tick single most appropriate)* ☐ 1 ☐ 2 ☐ 3 ☐ 4 ☐ Unknown/already intubated

VII. VENTILATION			
1	**Ventilation type:**<br>*(choose single most appropriate)*<br>☐ **Spontaneous ventilation**<br>☐ **Assisted ventilation**<br>☐ **Controlled ventilation**	1.1 *If controlled ventilation specify: (tick single most appropriate)*<br>☐ Volume controlled (VC)<br>☐ Pressure controlled (PC)<br>☐ Pressure regulated volume controlled (PRVC)<br>☐ High Frequency Oxillatory Ventilation (HFOV)	
		1.1.1 *For VC, PC, PRVC, please specify if initial setting is available?*<br>☐ No ☐ Yes	*If yes, specify*<br>1.1.1.1 *PIP* |__|__| cm/H_2O [0-99]<br>1.1.1.2 *PEEP* |__|__| cm/H_2O [0-20]<br>1.1.1.3.FiO_2 |__|.|__|__| [0.21-1.0]

VIII. END OF ANAESTHESIA/PROCEDURE TIMING		
1	**Date end of surgery/procedure:**	|__|__|- |__|__|__| - |__|__|__|__| [>=01-Mar-2016]
2	**Time End of surgery/procedure:**	Hours |__|__|[0-23] Minutes|__|__| [0-59]
3	**Date end of anaesthesia:**	|__|__|- |__|__|__| - |__|__|__|__| [>=01-Mar-2016]
4	**Time End of anaesthesia:**	Hours |__|__|[0-23] Minutes|__|__| [0-59]

IX. PERIOPERATIVE INTERVENTIONS

For definitions of Interventions for critical events please refer to last page of CRF

1	**Have you performed a medical intervention/treatment IN RESPONSE to a critical event?**	☐ No, go to Follow-up data<br>☐ Yes, complete the perioperative interventions section

If yes, fill in all the appropriate sections:
Section I AIRWAY MANAGEMENT
Section II OXYGENATION
Section III ALVEOLAR VENTILATION
Section IV METABOLIC
Section V CARDIOVASCULAR CONDITION
Section VI BODY TEMPERATURE
Section VII BRAIN OXYGENATION
Section VIII ANAEMIA

I INTERVENTION FOR DIFFICULT AIRWAY MANAGEMENT *defined as more than 2 unsuccessful attempts of intubation by direct laryngoscopy, which require alternative strategies for achieving successful intubation.*

1	**Has difficult AIRWAY MANAGEMENT, which needed intervention(s), occurred?**	☐ No<br>☐ Yes (fill in all sub-items)
**1.1 Specify Intervention(s) for difficult intubation:** *(tick all that apply)*<br>☐ Change of laryngoscope blades<br>☐ Supra-glottic airway device<br>☐ Use of video-assisted intubation<br>☐ Use of air-track<br>☐ Use of stylet or bougie<br>☐ Use of fiberoptic bronchoscopy<br>☐ Help from ENT colleague or 2nd senior anaesthesiologist<br>☐ Emergency tracheostomy<br>☐ Blind intubation<br>☐ Other		1.1.1 *If other, specify:*
**1.2 Was the difficult intubation an unplanned occurrence?**		☐ No ☐ Yes
**1.3 Was it associated with difficult face-mask ventilation?**		☐ No ☐ Yes
**1.4 Was a significant drop in oxygenation associated?**		☐ No ☐ Yes
**1.5 Was significant bradycardia associated?**		☐ No ☐ Yes
**1.6 Number of attempts until successful intubation:**		|__|__| [3-20]
**1.7 Outcome of event:**<br>*(tick single most appropriate)*	☐ Successful intubation<br>☐ Unsuccessful intubation, procedure performed under face or laryngeal mask<br>☐ Unable to intubate, patient woken up from anaesthesia	

**II OXYGENATION: Intervention for hypoxaemia** *defined as an action or a pharmacological treatment aimed at improving poor oxygenation and triggered by low SpO_2 and/or PaO_2.*			
2	**Has POOR OXYGENATION, which needed intervention(s), occurred?**	☐ No<br>☐ Yes (fill in all sub-items)	
2.1	**Specify Intervention(s) for improving oxygenation:** *(tick all that apply)*<br>☐ Emergency/unplanned intubation (if patient previously not intubated)<br>☐ Change/repositioning of an accidentally dislocated or obstructed tracheal tube<br>☐ Need for FiO_2 higher than for routine practice (or persistent need of FiO_2 = 1)<br>☐ Need for PEEP higher than for routine practice<br>☐ Need for prolonged manual ventilation in already intubated/ventilated patient<br>☐ Recruiting manoeuvre(s)<br>☐ Switch from conventional to non-conventional ventilation (HFOV)<br>☐ Drainage of an acute pneumothorax<br>☐ Pharmacological treatment of laryngospasm<br>☐ Pharmacological treatment of bronchospasm<br>☐ Other	2.1.1 *If other, specify:*	
2.2	**Time of occurrence of hypoxaemia** *(only if more than one, tick all that apply)*<br>☐ Induction ☐ Maintenance ☐ Awakening ☐ PACU		
2.3	**Number of poor oxygenation events that required intervention(s) during anaesthesia** *(tick single most appropriate)*<br>☐ Once ☐ Twice ☐ Three times or more, or persistent poor oxygenation		
2.4	**SpO_2 threshold that triggered intervention** *(choose single -refer to the most severe, if more than one):*<br>☐ < 90% ☐ < 85% ☐ < 80%		

2.5	**PaO_2 value that triggered intervention: |__|__|__|** *Specify units used:*<br>☐ Not Available<br>☐ *kPa* [0-100]<br>☐ *mmHg* [0-999]	
2.6	**provide approximate duration of hypoxaemia**:<br>**|__|__|__|** min. [1-999] ☐ Not Available	
2.7	**Outcome of event?** *(tick single most appropriate)*	☐ Successful - oxygenation improved<br>☐ Persistent Hypoxaemia<br>☐ Deterioration of oxygenation despite specific intervention(s)
2.8	**Was the poor oxygenation accompanied by a significant change in rSO_2 by NIRS?**	☐ No<br>☐ Yes. *If yes, fill in section VII (Brain oxygenation).*<br>☐ NIRS not available
2.9	**Was hypoxaemia associated with hypoventilation (hypercapnia)**	☐ Yes. *If yes, fill in section III (Intervention for HYPO or HYPERcapnia)*<br>☐ No
**III Intervention for HYPO or HYPERcapnia (without associated hypoxaemia)** *defined as an action to correct an alteration in End-tidal carbon dioxide ($ETCO_2$) and/or arterial/venous blood CO_2* level		
3	**Has altered CO_2, which needed intervention(s), occurred?**	☐ No<br>☐ Yes (fill in all sub-items)
3.1	**Specify Intervention(s) for improving alveolar ventilation**<br>☐ Change of ventilation modality<br>☐ Change of airway device (from face mask/SGAW to ETT)<br>☐ Change of the dead-space<br>☐ Need for Peak Inspiratory Pressure higher than for routine practice<br>☐ Prolonged/persistent manual ventilation<br>☐ Alveolar recruitment manoeuvre(s)<br>☐ Other	3.1.1 *If other, specify:*
3.2	**Number of interventions during anaesthesia:**<br>☐ Once ☐ Twice ☐ Three times and more, or persistent inadequate alveolar ventilation	

3.3	**$ETCO_2$ threshold that triggered intervention (refers to the most severe, if more than one):** *Specify units used:* |__|__|__| ☐ *kPa* [0-100] ☐ *mmHg* [0-999]	
3.4	**$PaCO_2$ value that triggered intervention:** *Specify units used:* |__|__|__| ☐ *kPa* [0-100] ☐ *mmHg* [0-999] ☐ Not available	
3.5	**Outcome of event?** *(tick single most appropriate)* ☐ Successful/ improvement ☐ Persistent difficult ventilation	**3.6 Were the CO_2 changes accompanied by a change in rSO2 by NIRS?** ☐ No ☐ Yes. *If yes, fill in section VII (Brain oxygenation).* ☐ NIRS not available
**IV METABOLIC Intervention** *defined as an action for correcting high/low blood glucose and/or Na^+:*		
4	**Have you performed treatment(s) for high/low glucose and/or Na^+?**	☐ No ☐ Yes (fill in all sub-items)
4.1	**Specify Intervention(s) for high/low glucose:** *(tick all that apply)*	☐ Administration of glucose (iv or continuous infusion) ☐ Stop i.v. glucose or fluids containing glucose ☐ Onset of insulin treatment
4.2	**Specify Intervention(s) for high/low Na^+:** *(tick all that apply)*	☐ Administration of additional Na+ (fluids or electrolytes) ☐ Stop i.v. glucose or hypotonic solutions
4.3	**What was the <u>glucose</u> and/or <u>Na^+</u> value(s) that triggered intervention(s):**	*Select units used:* ☐ Glucose |__|__|__| ☐ mMol/L [0-50] ☐ mg/dL [0-999] ☐ Na^+ |__|__|__|. |__| ☐ mMol/L [99-199] ☐ mEq/L [99.9-199.9]
4.4	**Outcome of event?** *(tick single most appropriate)*	☐ Successful - parameter corrected ☐ Persistent disorder
**V Intervention for CARDIOVASCULAR INSTABILITY** *defined as an intervention or a medical treatment to control cardio-vascular instability. This clinical condition can be triggered by the occurrence of hypo- or hyper-tension and/or cardiac rhythm disturbances on the ECG.*		
5	**Has CARDIOVASCULAR INSTABILITY, which needed intervention(s), occurred?**	☐ No ☐ Yes (fill in all sub-items)
5.1	**Was the intervention/treatment based on blood pressure:** ☐ No ☐ Yes	
5.2	**Number of interventions during anaesthesia:** ☐ Once ☐ Twice ☐ Three times and more	
5.3	**Specify Intervention(s) based on Blood Pressure and/or Cardiac Output:** ***Tick all that apply.*** ☐ Bolus of > 20 ml/kg of crystalloids (also 2 x 10 ml/kg) ☐ Bolus of > 10 ml/kg of albumin ☐ Bolus of > 10 ml/kg of other colloids ☐ Administration of Fresh Frozen Plasma for hypovolemia leading to C-V instability ☐ Administration of Packed Red Cells for C-V instability ☐ Other 5.3.1 *If other, specify:*	
5.4	**Blood pressure value that triggered intervention:** *Choose the one that have triggered your intervention:* ☐ *Systolic*	5.4.1 *Indicate value:* |__|__|__| mmHg [0-999]

	☐ *Mean*<br>☐ *Diastolic*	
**5.5**	**Total volume given as boluses with the aim to normalise blood pressure (do not include volume of fluids for maintenance):**	*Indicate value:*<br>|__|__|__| ml/kg [10-999]
**5.6**	**Time of occurrence** *(tick all that apply, only if more than one episode):*	☐ Induction<br>☐ Maintenance<br>☐ Whilst in PACU
**5.7**	**Was one or more of the following drugs given for BLOOD PRESSURE or CARDIAC OUTPUT instability (report only if drug(s) are given in response to a critical event; do not report if drugs are given as part of "usual protocol"):**	

5.7.1 Ephedrine ☐ No ☐ Yes	5.7.6 Dobutamine ☐ No ☐ Yes
5.7.2 Phenylephrine ☐ No ☐ Yes	5.7.7 Epinephrine/Adrenaline ☐ No ☐ Yes
5.7.3 Noradrenaline/Norepinephrine ☐ No ☐ Yes	5.7.8 Milrinone ☐ No ☐ Yes
5.7.4 Dopamine ☐ No ☐ Yes	5.7.9 Levosimendan ☐ No ☐ Yes
5.7.5 Nitroglycerine ☐ No ☐ Yes	5.7.10 Nitroprusside ☐ No ☐ Yes

**5.8**	**Blood pressure value that triggered DRUG administration:**<br>*Choose the one that have triggered the intervention:*<br>☐ *Systolic* ☐ *Mean* ☐ *Diastolic*	*5.8.1 Indicate value:*<br>|__|__|__| mmHg [0-999]
**5.9**	**Time of occurrence**<br>*(tick all that apply, only if more than one episode):*	☐ Induction<br>☐ Maintenance<br>☐ Whilst in PACU
**5.10**	**Was the intervention/treatment based on ECG disturbance:**	☐ No<br>☐ Yes
**5.11**	**If YES, specify Intervention(s) triggered by heart rate/ECG disturbances:**	☐ Atropine i.v. (ONLY for treatment, not for prevention)<br>☐ Glycopyrolate i.v. (ONLY for treatment, not for prevention)<br>☐ Epinephrine<br>☐ Ca++<br>☐ Mg++<br>☐ Lidocaine i.v.<br>☐ Amiodarone i.v.<br>☐ Electric defibrillation<br>☐ External pacing<br>☐ Chest compression < 1 min.<br>☐ Cardiopulmonary resuscitation (CPR)<br>☐ Other<br>5.11.1 *If other, specify:*
**5.12**	**If intervention was based on heart rate or ECG disturbance, please specify threshold value that triggered intervention (zero if cardiac arrest):**	|__|__|__| beats/min. [0-300]
**5.13**	**Approximate duration of instability:**	|__|__|__| min. [1-999]
**5.14**	**Time of occurrence** *(tick all that apply, only if more than one episodes):*	☐ Induction<br>☐ Maintenance<br>☐ Whilst in PACU

**5.15**	**Outcome of event:**	☐ Successful treatment<br>☐ Persistent cardiovascular instability
**5.16**	**Was the haemodynamic instability accompanied by a change in rSO_2 by NIRS?**	☐ No<br>☐ Yes. *If yes, fill in section VII (Brain oxygenation.*<br>☐ NIRS not available
**VI Intervention for high/low BODY TEMPERATURE**<br>*defined as an intervention for core body temperature derangement, in either direction (hypo/hyper).*		
**6**	**Has BODY TEMPERATURE ALTERATION which needed intervention(s), occurred?**	☐ No<br>☐ Yes (fill all sub-items)
**6.1**	**Specify interventions triggered by body temperature:**	☐ New onset of warming fluids *(if not already in use)*<br>☐ Active warming with blanket *(if not already in use)*<br>☐ Cooling fluids<br>☐ Active cooling body<br>☐ Other<br>6.1.1 *If other, specify:*
**6.2**	**If yes, specify the threshold trigger:**<br>*(tick single most appropriate)*	6.2.1 Location of probe:<br>☐ Oesophageal<br>☐ Rectal<br>☐ Cutaneous<br>6.2.2 Value of trigger:<br>|__|__| °C [30-45]
**6.3**	**Outcome of event:** *(tick all that apply)*	☐ Uneventful - successful treatment<br>☐ Cardiovascular instability<br>☐ Coagulopathy

**VII BRAIN OXYGENATION Intervention** *Defined as: when brain oxygenation with NIRS monitoring is part of clinical care, the occurrence of low rSO_2 and/or a drop in rSO_2. Any action or medical treatment to increase brain oxygenation will be also reported.*		
**7**	**Has any low rSO_2 and/or drop in regional cerebral oxygenation (rSO_2) occurred?**	☐ No ☐ Yes ☐ NIRS was not available
**7.1**	**Specify Interventions triggered by NIRS monitoring:**	☐ None<br>☐ Alteration in ventilation<br>☐ Intervention for systemic blood pressure<br>☐ Intervention for oxygenation<br>☐ Intervention for haemoglobin<br>☐ Intervention for cardiac output (Inotropes or via CPB in cardiac surgery)<br>☐ Other 7.1.1 *If other, specify:*
**7.2**	**If intervention, was this based on % drop or absolute value?**<br>☐ Absolute value (fill in 7.3) ☐ Percentage drop (fill in 7.4)	
**7.3**	**If absolute value specify rSO_2 value:**<br>**|__|__|__| %** [0-100]	**7.4 If percentage specify the percentage drop from baseline:**<br>**|__|__|__|%** [20-100]
**VIII**	**Packed Red Cells administered?** (if PRC given for cardiovascular instability, please refers to section V)	
**8**	**Were Packed Red Cells administered for ANAEMIA as primary reason?**<br>☐ No<br>☐ Yes	8.1 *If yes, specify* haemoglobin level that triggered transfusion,<br>|__|__|.|__| *Specify units used:* ☐ g/dL [0.0-20.0] ☐ mMol/L [0.0-20.0]<br>8.2 Total volume administered intraoperatively : |__|__|__| ml

X. END OF ANAESTHESIA AND UP TO 120 MINUTES (or until PACU discharge)

1	**Where was the child transferred after PACU discharge?**<br>☐ PICU/NICU ☐ Intermediate Care/High Dependency Unit ☐ Ward ☐ Other	
1.1	**If PICU/NICU, was admission UNPLANNED?**<br>☐ No<br>☐ Yes	
1.1.1	**If YES (unplanned), is PICU/NICU admission due to above reported critical event(s):**<br>☐ No<br>☐ Yes	
1.2	**Was the child left intubated at PICU/NICU admission?**<br>☐ No<br>☐ Yes (fill in item 2)	
1.2.1	**If left INTUBATED, was delayed extubation UNPLANNED and/or related to the above reported critical event(s)?**<br>☐ No<br>☐ Yes	
2	**Relevant postoperative bleeding which needed surgical revision prior to PACU discharge?**<br>☐ No<br>☐ Yes	
3	**Need for postoperative ECMO?**<br>☐ No<br>☐ Yes	
4	**Need to leave chest open (only for cardiac surgery)?**<br>☐ No<br>☐ Yes	
5	**In-hospital overnight admission while scheduled as outpatient?**<br>☐ No ☐ Yes	**5.1 If YES, was delay in hospital discharge due to above reported critical event(s)?**<br>☐ No ☐ Yes

CRF 3-A: FOLLOW UP AT DAY 30 (Not repeated = 1 per patient)		
Complete 30 days after the LAST anaesthesia (if multiple occurrence) with data from the medical record. This is mandatory for all patients		
**1**	**Was follow-up made after 30 days (+/- 2 days), using the original medical record?**<br>☐ No ☐ Yes If no, END OF QUESTIONNAIRE	1.1 If yes, date of Day 30 follow-up |__|__| - |__|__|__| - |__|__|__|__| [>=01-Mar-2016]
**2**	**Patient Status at Day 30**<br>*(tick single most appropriate)*	☐ Discharged to home<br>☐ Discharged to another hospital<br>☐ Still in hospital<br>☐ Still in ICU<br>☐ Death
**2.1**	**Date of discharge or death:**<br>**|__|__| - |__|__|__| -|__|__|__|__|** dd/Mmm/yyyy [>=01-Mar-2016]	**2.2 If death, suspected cause:**
**3**	**Total day(s) in PICU/NICU (zero never in ICU):** |__|__| days [0-30]	
**4**	**Total day(s) on ventilation (zero never ventilated):** |__|__| days [0-30]	
**5**	**Was the patient admitted/re-admitted to PICU/NICU during the 30 days of follow-up (as a separate event from the immediate postoperative period)?** ☐ No ☐ Yes	
**6**	**MORBIDITY at Day 30 (or day of discharge):**<br>Has the child experienced any complication(s) in the 30 days following anaesthesia?	*Please fill yes or no ONLY if you have access to the medical file / if no access, please tick unknown*<br>☐ No<br>☐ Yes (If yes, choose the appropriate complication(s) from 6.1 to 6.6)<br>☐ Unknown
**6.1**	**BRAIN/CNS complication?**<br>☐ No ☐ Yes ☐ info not Available	6.1.1 *If yes, tick all that apply:*<br>☐ Hypertonia (new onset)<br>☐ Hypotonia (new onset)<br>☐ Intracranial bleeding (ultrasound, CT, or MRI)<br>☐ Intracranial ischaemia (ultrasound, CT, or MRI)<br>☐ Occurrence of seizures (clinically or EEG)
**6.2**	**SURGICAL complication?**<br>☐ No ☐ Yes ☐ info not Available	6.2.1 *If yes, tick all that apply:*<br>☐ Re-operation for unsuccessful or complicated first surgery<br>☐ Severe surgical site infection with new onset of antibiotics<br>☐ Need for prolonged parenteral nutrition do to surgical complication
**6.3**	**RESPIRATORY complication?**<br>☐ No ☐ Yes ☐ info not Available	6.3.1 *If yes, tick all that apply:*<br>☐ ECMO (Extra Corporeal Membrane Oxygenation)<br>☐ Failure of weaning with prolonged ventilator support<br>☐ Need for re-intubation after being extubated<br>☐ Pleural effusion<br>☐ Pneumonia<br>☐ Pneumothorax
**6.4**	**CARDIO-VASCULAR complication?**<br>☐ No ☐ Yes ☐ info not Available	6.4.1 *If yes, tick all that apply:*<br>☐ Arrhythmia<br>☐ Episode(s) of Cardiac Arrest<br>☐ Cardiac Ischaemia (elevated troponine)<br>☐ ECMO (Extra Corporeal Membrane Oxygenation)<br>☐ Arterial/Venous Embolism<br>☐ Inotropes-Vasopressors needed (after 1st day)<br>☐ Venous Thrombosis (on central venous line)
**6.5**	**LIVER FAILURE?**<br>☐ No ☐ Yes ☐ info not Available	6.5.1 *If yes, tick all that apply:*<br>☐ Coagulation Disorders (Increase in INR >2)<br>☐ Increase in serum bilirubin >300 micromol/L (>10 mg/dL)
**6.6**	**RENAL INSUFFICIENCY?**<br>☐ No ☐ Yes ☐ info not Available	6.6.1 *If yes, tick all that apply:*<br>☐ Continuous renal replacement therapy (CRRT)<br>☐ Increase creatinine levels necessitating adaptation of medications<br>☐ Peritoneal dialysis

**CRF 3-B: FOLLOW UP AT DAY 90**<br>**(Non-Repetitive = 1 per patient => *Should occur only ONCE at 90 days (+/-2 days) after patient's LAST NECTARINE anaesthesia/procedure)***		
If patient still at hospital complete with MEDICAL RECORD; if patient is discharged before day 90 to be done with telephone CALL or FACE TO FACE visit)		
**1**	**Was a follow-up made after 90 days (+/- 2 days)**<br>☐ No ☐ Yes	If no, END OF QUESTIONNAIRE<br>1.1 *If yes, date of Day 90 follow-up*<br>|__|__| - |__|__|__| - |__|__|__|__| [>=01-Mar-2016]
**2**	**Patient Status at 90 days?**	*Please tick 1 of the following:*<br>☐ Alive – discharged home before day 30 (fill in 2.2)<br>☐ Alive – discharged home between 30 and 90 days (fill in 2.1 and 2.2)<br>☐ Alive - still in Hospital at 90 days (fill in 2.2)<br>☐ Death - between 30 and 90 days (fill in 2.3 and 2.4)
**2.1**	**Date of discharged (between 30 and 90 days):**	|__|__| - |__|__|__| - |__|__|__|__| [>=01-Mar-2016]
**2.2**	**Any hospital re-admission until the 90-day follow-up?**<br>☐ No ☐ Yes	*2.3.1 If YES, specify* reason for hospital readmission*:*
**2.3**	**Date of death (between 30 and 90 days):**	|__|__| - |__|__|__| - |__|__|__|__| [>=01-Mar-2016]
**2.4**	**If deceased at 90 days, suspected cause of death:**	*Briefly describe cause of death:*

Appendix 3 – Definitions and list of interventions:

Intervention is an action/medical treatment which is performed **in response to a critical event and/or a physiological parameter derangement** as judged by the anaesthesia team.

The medical treatment given for preventing a critical event will not be considered as an intervention.

**1. Interventions for Difficult airway management:**	Defined as more than 2 unsuccessful attempts of intubation by direct laryngoscopy, which require alternative strategies.
**2. Interventions for Oxygenation:**	Defined as an action or a pharmacological treatment performed by the anaesthesia team aimed at improving a status of poor oxygenation. The specific intervention can be triggered by SpO_2 and/or PaO_2.
**3. Intervention for Alveolar ventilation:**	Defined as an action or a treatment performed by the anaesthesia team aimed at correcting an alteration in CO_2 levels (End-tidal-CO_2 and/or P-arterial/venous CO_2).
**4. Interventions for correcting Glycaemia, Na^+ levels:**	Defined as a treatment performed by the anaesthesia team aimed at correcting abnormal levels of blood glucose and/or Na, in either direction (hypo/hyper).
**5. Intervention for Cardiovascular instability:**	Defined as an intervention or a medical treatment to control cardio-vascular instability. This clinical condition can be triggered by the occurrence of hypo- hypertension and/or cardiac rhythm disturbances on the ECG.
**6. Intervention for correcting body temperature:**	Defined as an intervention for core body temperature derangement, in either direction (hypo/hyper).
**7. Interventions induced by poor Brain oxygenation:**	Defined as the occurrence of low rSO_2 and/or a drop in rSO_2 when brain oxygenation with NIRS monitoring is part of clinical care. Any action or medical treatment to increase brain oxygenation will also be reported.
**8. Packed Red Cells transfusion for Anaemia:**	This section will be filled in in case of Packed Red Cell transfusion for intraoperative anaemia as primary reason, and not for cardiovascular instability (in this case refers to section 5). The Hb level that has triggered the transfusion will be reported.
